# A systematic review of faculty development programs based on the Harden teacher’s role framework model

**DOI:** 10.1186/s12909-023-04863-4

**Published:** 2023-11-30

**Authors:** Mahmoud Kohan, Tahereh Changiz, Nikoo Yamani

**Affiliations:** 1 Department of Medical Education, Isfahan University of Medical Sciences, Isfahan, Iran; 2 Department of Medical Education, Medical Education Research Center, Isfahan University of Medical Sciences, Isfahan, Iran; 3 Medical Education Research Center, Medical Education Development Center, Isfahan University of Medical Sciences and Health Services, Isfahan, Iran

**Keywords:** Faculty development, Program evaluation, Faculty, Role, Competency-based education

## Abstract

**Background:**

Despite the changing roles of faculty in the health professions over the past two decades, none of the reviews has been paid enough attention to the impact of the faculty development programs on these roles. The objective of this review is to synthesize the existing evidence that addresses the questions: “What are the types and outcomes of faculty development programs based on the Harden teachers’ role framework and which of the areas described by Harden and Crosby are the authors referring to?”

**Methods:**

This review was conducted according to the guidance for Preferred Reporting Items for Systematic Reviews and Meta-Analyses (PRISMA) framework. In 2020, a literature search was conducted in MEDLINE/PubMed, Scopus, ERIC, ScienceDirect, Google Scholar, Magiran and SID databases. The review included 119 studies (between 1990 and 2020) that met the review criteria. Data were extracted using a modified coding sheet. We used the modified Kirkpatrick model to assess the educational outcomes of faculty development programs.

**Results:**

The majority of faculty development programs were workshops (33.61%) with various durations. Most programs focused on the domain of information provider and coach (76.47%), followed by the facilitator of learning and mentor (53.78%) and assessor and diagnostician (37.81%). Only five faculty development programs focused on the domain of role model. The majority (83.19%) of outcomes reported were at level 2B, level 1 (73.95%) and level 2A (71.42%). Gains in knowledge and skills related to teaching methods and student assessment were frequently noted. Behavior changes included enhanced teaching performance, development of new educational curricula and programs, improved feedback and evaluation processes, new leadership positions, increased academic output and career development. The impact on the organizational practice continued to be underexplored.

**Conclusion:**

Based on the review findings, broadening the scope of faculty development programs beyond the traditional roles of the faculty members by utilizing a competency-based framework for developing a comprehensive faculty development program is recommended. Attention to individualized form of faculty development programs and incorporating more informal approaches into the design and delivery of faculty development programs is also needed.

**Supplementary Information:**

The online version contains supplementary material available at 10.1186/s12909-023-04863-4.

## Background

**Fig. 1:**
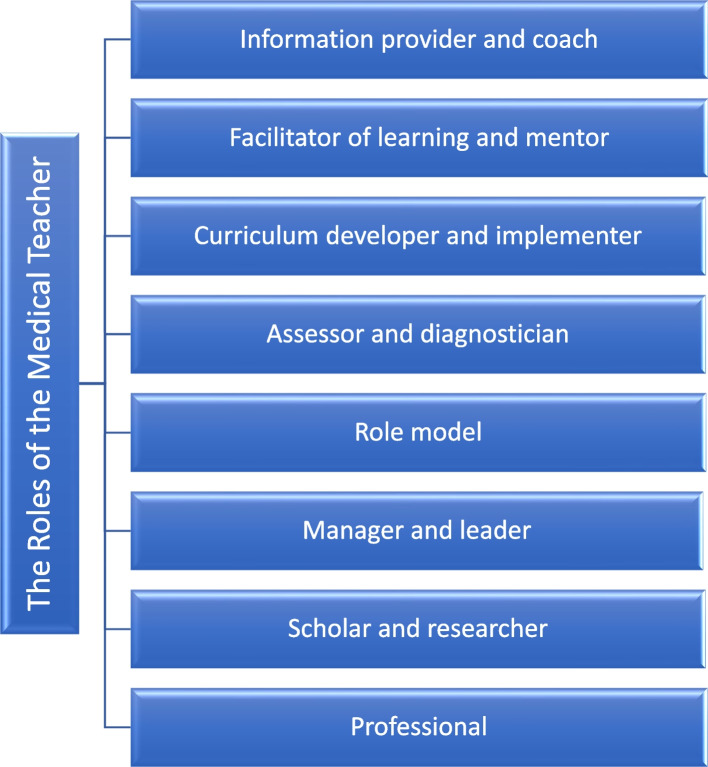
The eight roles of the medical teachers

Faculty development has become an integral part of organizational development [1] and can play an important role in promoting organizational change [2]. As noted by Wilkerson and Irby [3], “Academic vitality is dependent upon faculty members’ interest and expertise; faculty development has a critical role to play in promoting academic excellence and innovation, and it is a tool for improving the educational vitality of our institutions through attention to the competencies needed by individual teachers and to the institutional policies required to promote academic excellence”. This notion is further supported by the work of Harden and Crosby [4], who described and were further expanded the eight roles of the medical teacher (information provider and coach, facilitator of learning and mentor, curriculum developer and implementer, assessor and diagnostician, role model, manager and leader, scholar and researcher and professional) in the education programme (Fig. 1). This teachers’ role framework can be considered as a guide for designing and evaluating faculty development programs, as described by Harden and Crosby [4], in that it provides evidence about which faculty’ roles covered by the programs and identifies the needs for faculty development programs [4]. Moreover, considering this competency-based framework in the design and evaluation of faculty development programs can play an important role in expanding our approaches to faculty development programs and would help to inform both content and desired outcomes [2].

To date, few publications have reviewed the impact and outcome of faculty development programs in the health professions education. Despite the emergence of outcome-based education and its impact on faculty development programs [5] on the one hand, and the introduction of medical teachers’ role framework by Harden and Crosby [4] on the other, none of the reviews followed this teachers’ role framework. In 2006 and 2016, Steinert et al. reviewed the literature on faculty development initiatives designed to improve teaching effectiveness in medical education and concluded that despite the usefulness of faculty development programs for participants and changes in their learning and behavior, further research to explore outcomes at the individual and organizational levels is needed. These reviews were limited to faculty development designed to enhance the effectiveness of teaching in medicine. They suggested that it would now be useful to update these reviews and to carry out a similar review of faculty development focusing on other faculty roles [6, 7]. Johnston et al. [8] reviewed 70 articles on faculty development initiatives in academic dentistry and pointed to the need for more research in more areas and using additional methodologies. Bilal et al. [9] conducted a systematic review of studies, published during 2003–2016, to explore the effectiveness of faculty development programs on medical and allied health faculty’s professional development. They highlighted that there is no well-structured theoretical framework for faculty development programs that can be incorporated across institutions. More recently, Behar-Horenstein et al. [10] reviewed the literature (published between 2006 and 2018) on faculty development initiatives in the health professions and recommended that future faculty development studies include pre-test and post-test measures with control and treatment groups or implement time series studies to assess the impact of the intervention beyond the conclusion of faculty development program. We decided to conduct this review for several reasons. Firstly, we believed that this 30-year review would allow us to describe the evolution of faculty development programs in the past three decades. Secondly, we wanted to identify emerging trends and articulate a sound theoretical basis to help advance the practice. Thirdly, despite the changing roles of faculty in the health professions over the past two decades, none of the reviews has been paid enough attention to the impact of the faculty development programs on these roles. Fourthly, given the expanding number of roles of faculty in the health professions, there has been no comprehensive systematic review of faculty development programs to target all health professions teachers’ roles. Lastly, we wanted to address limitations and recommendations identified by previous reviews.

**Fig. 2:**
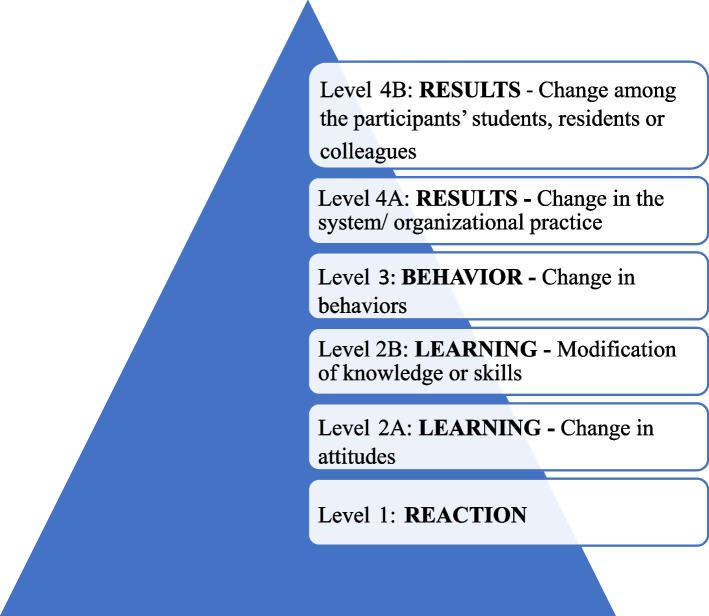
Kirkpatrick’s model for evaluating educational outcomes

Notably, our study is the first systematic review of faculty development programs based on the Harden teachers’ role framework model. In addition, we appraised the quality of the studies using a reliable tool for appraising methodological quality of medical education research, the Medical Education Research Study Quality Instrument (MERSQI) [11]. Moreover, we used the modified Kirkpatrick model (Fig. 2) to assess the educational outcomes of faculty development programs. This model was adopted by the BEME (Best Evidence Medical Education) Collaboration and includes students, residents and colleagues (instead of patients) at level 4B [7]. It is also noteworthy that in the present study, in addition to international databases, national databases were also searched from 1990 to 2020. Given the potential importance of faculty development programs in the health professions, the objective of this review is to determine the types and outcomes of faculty development programs based on the Harden teachers’ role framework model and to identify, or explain, to which of the areas described by Harden and Crosby the authors are referring. More specifically, this review addressed the following research questions:

- What are the types and duration of faculty development programs designed to prepare faculty members for their various roles in health sciences education?
- Which of the areas, or faculty’ roles, described by Harden and Crosby are the authors referring to?
- What are the impacts of faculty development programs on the knowledge, attitudes and skills of faculty members in health sciences education, and on the institutions in which they work?

We hope that such a review of the literature and a synthesis of quantitative and qualitative studies will help to conceptualize our knowledge of the field and guide educators interested in the design, development, and evaluation of faculty development.

## Methods

This systematic review was conducted according to the guidance for Preferred Reporting Items for Systematic Reviews and Meta-Analyses (PRISMA) framework suggested by Moher et al. [12] in January 2020 to explore the types and outcomes of faculty development programs designed to prepare faculty members for their various roles in health sciences education based on the Harden teachers’ role framework model. The review was a part of a doctoral dissertation performed to fulfil the requirements of a Ph.D. (Doctor of Philosophy) program in medical education at Isfahan University of Medical Sciences and Health Services. The study was approved by the Institutional Research Ethics Committee of Medical University of Isfahan.

### Information sources and search strategy

In January 2020, the authors conducted an extensive review of published articles between 1990 and 2020 to identify all potentially relative studies. A literature search was conducted in MEDLINE/PubMed (Last searched on February 29, 2020), Scopus (Last searched on April 30, 2020), ERIC (Last searched on June 30, 2020), ScienceDirect (Last searched on August 30, 2020), Google Scholar (Last searched on October 30, 2020), Magiran (Last searched on November 30, 2020) and SID (Scientific Information Database, Last searched on December 30, 2020) using the following keywords: “faculty development”; “faculty empowerment”; “faculty training”; “faculty education”; “faculty promotion”; “teacher education”; “teacher training”; “teacher improvement”; “teacher promotion”; “in-service training”; “in-service teacher education”; “professional development”; “medical education”; “program evaluation”; “medical teacher”; “health sciences educator/education”; “medical faculty”; “nursing faculty”; “dental faculty”; “pharmacy faculty” and “clinical teacher”. Keywords were combined with the Boolean operators AND/OR. Although the search was not limited by country of practice, the search was conducted with limitation on publication year, language, study design and full text availability. Search results limited to full text empirical studies published in English or Persian during 1990–2020. We chose these databases because they cover faculty development programs in the health sciences education. A copy of the search strategy used within MEDLINE/PubMed is provided in Appendix 1 (see Additional file 1). In addition, we conducted an extensive review of published articles in medical education core journals. The journals searched included: Medical Teacher, Medical Education, Academic Medicine, Teaching and Learning in Medicine, The Clinical teacher, Medical Science Educator, International Journal of Medical Education, Advances in Health Sciences Education and The Journal of Faculty Development. We also searched reference lists of all review articles.

### Inclusion/exclusion criteria

The following criteria guided the selection of studies for this review:

- *Study focus*: Based on the review questions, all types of faculty development programs, of whatever duration, designed to prepare faculty members for their various roles in health sciences education were included. Studies not relating to faculty development programs in the health professions were excluded.
- *Study population*: We included studies that involved faculty development programs for both basic science and clinical faculty members in all disciplines of health sciences. Any study involving the development of staff and students was excluded.
- *Study design*: To be included, studies must have intervention and measurement (outcome data). Thus, only empirical studies with pre-test post-test design were included. Both quantitative and qualitative studies were included. Reviews and articles described general information about faculty development programs with no intervention or evaluation data were excluded. Additionally, empirical studies with post-test design only were excluded.
- *Year of publication*: We selected all qualitative and quantitative studies assessing faculty development programs from 1990 to 2020 for inclusion. The selection of articles for review was completed in 2020.
- *Language and country of practice*: Although the search was not limited by country of practice, the review was limited to studies published in English and Persian. Articles not written in the English or Persian language were excluded.

### Screening and study selection

**Fig. 3:**
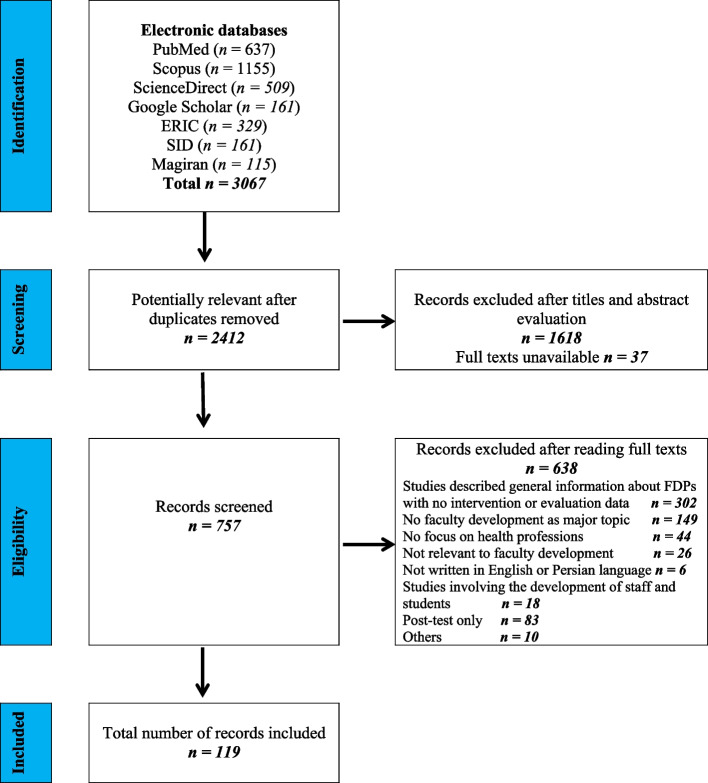
PRISMA flow chart of the systematic review

In total 3067 articles were identified through the initial search of the seven databases. The study screening was done by three authors (MK, NY and TC) in three stages: by title, then by abstract, and finally by full text review. From an initial 3067 articles, 2412 articles were potentially relevant after duplicates removed by one of three reviewers (MK). After removing duplicates, title and abstract screening was undertaken independently by two authors (MK or NY) with disagreements between them resolved by consensus. A third author (TC) was recruited to facilitate agreement when needed. This resulted in 757 articles being identified as potentially relevant and retrieved in full text for comprehensive review. Full-text screening was carried out by one of two authors (MK or NY) based on pre-determined eligibility criteria with any studies deemed ineligible cross-checked by the third author (TC). Of the 757 full papers that were screened, 638 studies were excluded as they did not meet the inclusion criteria for the review. Finally, a total of 119 studies met the inclusion criteria and were submitted to data extraction (Fig. 3).

### Data extraction and summary of findings

Relevant information was extracted from each included studies using a coding sheet, which was developed from previous systematic review work (BEME Guide No. 8). The applicability of the coding sheet was assessed in the original study by the pilot study and the Faculty Development Topic Review Group members’ research experience [7]. The coding sheet, which was based on the original prototype provided by the BEME Guide No. 8, was modified to data extraction. Data were extracted included following components: authors and publication years, country and institution, type of program or intervention and duration, participants, faculty roles covered by the program, study design, outcomes, outcome level and study quality (MERSQI) score. The coding sheet is provided in Appendix 2 (see Additional file 2). Data extraction was conducted by MK checked by a second author (NY). Where necessary, the third reviewer (TC) assisted in resolving differences. To identify to which of the areas, or faculty^,^ roles, described by Harden and Crosby the authors are referring, two authors (MK and NY) separately read the research articles. Thereafter, they assigned the faculty development programs to one or more of Harden’s eight domains. Discrepancies in judgment between the two authors were resolved through discussion. The third author (TC) was recruited to facilitate agreement when needed. Once the coding sheet was completed, the extracted data were grouped for the synthesis stage based on the review objectives. This was done manually by the first author (MK) and checked by the second author (NY).

### Quality assessment

The quality of the studies was appraised using the Medical Education Research Study Quality Instrument (MERSQI). We chose this instrument because the MERSQI was developed to appraise the methodological quality of experimental, quasi-experimental, and observational studies in medical education research, typically in the process of a literature review of a field or topic in medical education [11]. The MERSQI evaluates study quality based on the following items: study design, sampling (institutions & response rate), type of data, validity evidence for evaluation instrument scores, data analysis (sophistication & appropriate) and outcome. Each item is scored on a scale of 1–3 and summed to determine a total score. The maximum score for each domain was 3, therefore the maximum MERSQI score is 18 with a potential range of 5–18.

## Results

For better collation and interpretation, review findings will be organized into four sections:

- *Overview of articles included in review*- which will be further divided into: publication years, country and institution.
- *Description of faculty development programs and expected outcomes*- which will be further divided into: type of program or intervention, duration, participants, outcomes and outcome level.
- *Eight domains of faculty roles covered by the faculty development programs according to Harden*- which will be further divided into: the teacher as an information provider and coach, the teacher as a facilitator of learning and mentor, the teacher as a curriculum developer and implementer, the teacher as an assessor and diagnostician, the teacher as a role model, the teacher as a manager and leader, the teacher as a scholar and researcher and the teacher as a professional.
- *Quality appraisal of the studies*- which will be further divided into: study design, sampling (institutions & response rate), type of data, validity evidence for evaluation instrument scores, data analysis (sophistication & appropriate) and outcome.

- *Overview of articles included in review*

Figure 3 provides an overview of the search strategy and the process of study selection. The characteristics of the 119 included studies, along with the quality assessment score, are summarised in Table S1 (see Additional file 3).

### Publication years

The year of publication for the 119 selected studies ranged from 1992 to 2019. The year with the highest number of published studies was 2017 (*n* = 12, 10.08%).

### Country and institution

Of the 119 articles reviewed, most of the studies were conducted in the USA (*n* = 86, 72.27%). Ninety two of the included studies (77.31%) were conducted by universities. Of these, 33 (35.86%) came from schools of medicine; Ten (10.86%) were conducted by colleges of Pharmacy; 8 (8.69%) were undertaken by schools of dentistry and two implemented in nursing schools. Table 1 shows the results of this section.

**Table 1:** General characteristics of the included studies

	***n*** **, total = 119**	**%**
**Publication years**		
1992-1996	4	3.4
1997-2001	5	4.2
2002-2006	25	21
2007-2011	13	10.9
2012-2016	46	38.7
2017-2020	26	21.8
**Country**		
USA	86	72.27
Canada	5	4.20
India	4	3.37
Iran	3	2.52
Germany	2	1.68
Nepal	2	1.68
Russia	2	1.68
Switzerland	2	1.68
Sweden	2	1.68
Bhutan	1	0.84
China	1	0.84
Chile	1	0.84
Japan	1	0.84
Mexico	1	0.84
Mongolia	1	0.84
Qatar	1	0.84
South Korea	1	0.84
Tanzania	1	0.84
Taiwan	1	0.84
UK	1	0.84
**Institution**		
Universities	92	77.31
Hospitals	10	8.40
Foundations and societies	17	14.29

(b) *Description of faculty development programs and expected outcomes*.

### Type of program or intervention

According to the included studies, the majority of faculty development programs were workshops (*n* = 40, 33.61%) with various durations. Nineteen (nearly 16% of faculty development programs) were described as a short course and sixteen (13.44%) as a seminar series. In six (5.04%) studies, the use of a mentorship program was described. There were three fellowship studies. Twenty nine (24.36%) were described as a longitudinal program and six fell under ‘other’, which included a journal club, OSTE (Objective Structured Teaching Exercise) and conferences.

### Duration

The duration of faculty development programs varied depending on the type of intervention and ranged from 20 min to six years. The duration of workshops ranged from one hour to six weeks, with a median duration of two days. The duration of the seminar series ranged from one and a half hours to seven weeks, with a median duration of twenty-eight hours. The short courses ranged from one hour to four weeks (with a median duration of one day), and the longitudinal programs ranged from seven weeks to five years with a median duration of one year. Mentorship programs ranged in duration from nine months to 4 years, with a median duration of two years. The duration of fellowship programs ranged from one year to six years.

### Participants

The 119 included studies involved a total of 7633 participants (sample sizes from 5 to 516). Studies were heterogeneous in terms of participant populations. The majority of faculty development programs (*n* = 64, 53.78%) involved faculty from multiple disciplines. Thirty five (29.41%) studies out of 119 occurred in medicine only. Studies in pharmacy (*n* = 10, 8.4%), dentistry (*n* = 8, 6.72%) and nursing (*n* = 2, 1.68%) constituted less than one fifth of the articles reviewed. In twelve (10.08%) studies, in addition to faculty members, residents also participated as participants. Twenty six (21.84%) faculty development programs were designed for both basic scientists and clinical faculty members. The majority of faculty development programs targeted clinical faculty members, with a predominance of interventions in family medicine and internal medicine.

### Outcomes and outcome level

We classified the reported outcomes using the Kirkpatrick’s model of educational outcomes. The majority (*n* = 99, 83.19%) of outcomes reported are at level 2B (modification of knowledge or skills). Only four studies [13–16] assessed change among the participants’ students, residents or colleagues (level 4B). Table 2 shows the summary of faculty development outcomes by Kirkpatrick level.

**Table 2:** Summary of faculty development outcomes by Kirkpatrick level*

Level of outcomes	%
Reaction	73.95
Learning	94.95
85/119 assessed changes in attitudes	
99/119 assessed changes in knowledge/skills	
Behavior	49.57
Results	15.96
19/119 assessed change in the system/ organizational practice	
4/119 assessed change among the participants’ students, residents or colleagues	

*Numbers may not equal 100% as some studies assessed outcomes in more than one way

(c) *Eight domains of faculty roles covered by the faculty development programs according to Harden*.

Summary of included studies based on Harden’s eight domains is presented in Table 3. As we anticipated, the majority (*n* = 79, 66.38%) of faculty development programs covered more than one domain of faculty roles; however, none of the programs covered all eight domains of faculty roles. Most of the articles focused on the domain of information provider and coach (76.47%), followed by the facilitator of learning and mentor (53.78%) and assessor and diagnostician (37.81%). We have detailed the study findings based on the Harden teacher’s role framework in Appendix 3 (see Additional file 4).

**Table 3:** Summary of included studies based on Harden’s eight domains

	**Information provider and coach**	**Facilitator of learning and mentor**	**Curriculum developer and implementer**	**Assessor and diagnostician**	**Role model**	**Manager and leader**	**Scholar and researcher**	**Professional**
**Frequency n (%)**	91 (76.47%)	64 (53.78%)	18 (15.12%)	45 (37.81%)	5 (4.2%)	19 (15.96%)	29 (24.36%)	24 (20.16%)
**Type of Intervention**								
Workshops	32 (35.16%)	24 (37.50%)	6 (33.33%)	18 (40%)	1 (20%)	7 (36.84%)	10 (34.48%)	4 (16.66%)
Short course	15 (16.48%)	9 (14.06%)	1 (5.55%)	6 (13.33%)	3 (60%)	2 (10.52%)	3 (10.34%)	4 (16.66%)
Seminar series	15(16.48%)	9 (14.06%)	3 (16.66%)	10 (22.22%)	0	3 (15.78%)	3 (10.34%)	3 (12.5%)
Mentorship	2 (2.19%)	3 (4.68%)	1 (5.55%)	0	0	0	3 (10.34%)	3 (12.5%)
Fellowship	2 (2.19%)	1 (1.56%)	1 (5.55%)	1 (2.22%)	0	1 (5.26%)	3 (10.34%)	1 (4.16%)
Longitudinal program	20 (21.97%)	17 (26.56%)	6 (33.33%)	9 (20%)	1 (20%)	6 (31.57%)	6 (20.68%)	9 (37.5%)
Other	5 (5.49%)	1 (1.56%)	0	1 (2.22%)	0	0	1 (3.44%)	0
**Study design**								
Single group	58 (63.73%)	37 (57.81%)	7 (38.88%)	26 (57.77%)	3 (60%)	9 (47.36%)	16 (55.17%)	15 (62.5%)
Cohort study	19 (20.87%)	17 (26.56%)	7 (38.88%)	12 (26.66%)	1 (20%)	7 (36.84%)	9 (31.03%)	6 (25%)
Nonrandomised controlled study	11 (12.08%)	9 (14.06%)	4 (22.22%)	6 (13.33%)	1 (20%)	3 (15.78%)	4 (13.79%)	3 (12.5%)
Randomized controlled study	3 (3.29%)	1 (1.56%)	0	1 (2.22%)	0	0	0	0
Qualitative methodology	1 (1.09%)	1 (1.56%)	0	0	0	0	0	1 (4.16%)
Mixed methodology	11 (12.08%)	9 (14.06%)	2 (11.11%)	7 (15.55%)	2 (40%)	1 (5.26%)	6 (20.68%)	5 (20.83%)
**Type of Data**								
Self-reported	50 (54.94%)	35 (54.68%)	9 (50%)	23 (51.11%)	2 (40%)	10 (52.63%)	18 (62.06%)	13 (54.16%)
Objective	3 (3.29%)	2 (3.12%)	1 (5.55%)	5 (11.11%)	0	0	0	0
Self-reported, Objective	38 (41.75%)	27 (42.18%)	8 (44.44%)	17 (37.77%)	3 (60%)	9 (47.36%)	11 (37.93%)	11 (45.83%)
**Level of outcomes**								
Level 1	70 (76.92%)	53 (82.81%)	14 (77.77%)	35 (77.77%)	4 (80%)	17 (89.47%)	23 (79.31%)	18 (75%)
Level 2A	68 (74.72%)	56 (87.50%)	15 (83.33%)	35 (77.77%)	5 (100%)	15 (78.94%)	21 (72.41%)	21 (87.5%)
Level 2B	76 (83.51%)	53 (82.81%)	15 (83.33%)	40 (88.88%)	4 (80%)	18 (94.73%)	26 (89.65%)	20 (83.33%)
Level 3	47 (51.64%)	31 (48.43%)	10 (55.55%)	28 (62.22%)	2 (40%)	12 (63.15%)	17 (58.62%)	14 (58.33%)
Level 4A	17 (18.68%)	13 (20.31%)	7 (38.88%)	9 (20%)	2 (40%)	7 (36.84%)	9 (31.03%)	7 (29.16%)
Level 4B	4 (4.39%)	2 (3.12%)	1 (5.55%)	1 (2.22%)	0	0	1 (3.44%)	1 (4.16%)

#### The teacher as an information provider and coach

With reference to Harden’s domains, the one most covered by the faculty development programs is an information provider and coach, which can be identified in 91 studies [13–103]. Pharmacy, nursing, medical, and dental faculty as well as basic science and clinical science teachers from different departments participated in these studies. The majority of outcomes reported were at level 2B (83.51%). The majority of faculty development programs led to significant increases in faculty members’ cognitive knowledge and skills of different aspects of the teaching-learning process and coaching such as educational concepts and principles, teaching strategies, methods and techniques. Participants also reported an improvement in their teaching and coaching skills such as teaching communication skills, clinical teaching, office-based teaching, giving effective feedback and promoting reflection.

#### The teacher as a facilitator of learning and mentor

Sixty four of faculty development programs focused on the domain of facilitator of learning and mentor [13, 16, 18, 23–27, 29, 30, 32, 37–42, 44, 45, 47–50, 52–62, 64, 66, 67, 69–73, 77, 78, 81–83, 85–88, 90, 93, 96, 97, 99, 100, 102–108]. Studies included faculty participants in medicine, nursing, pharmacy and dentistry and as well as basic science and clinical science teachers from different departments. The majority of outcomes reported were at level 2A (87.50%). Most programs led to self-reported changes in participants’ attitudes about and perceptions of teaching, learning and mentoring.

#### The teacher as a curriculum developer and implementer

Eighteen of faculty development programs focused on the domain of curriculum developer and implementer [14, 25, 44, 52–54, 58, 61, 79, 81, 83, 84, 87, 93, 100, 106, 109, 110]. Participants in these studies included community-based physician faculty, critical care faculty, academic podiatric physicians and faculty in medicine, nursing, pharmacy, dentistry, psychology, nutrition, audiology, physical therapy and occupational therapy as well as faculty from seven surgical and related disciplines and five medical subspecialties. The majority of outcomes reported were at level 2A and level 2B (83.33% each). Most programs led to significant changes in participants’ confidence for a wide range of academic skills such as curriculum design.

#### The teacher as an assessor and diagnostician

Forty-five of faculty development programs focused on the domain of assessor and diagnostician [15, 18, 20, 23, 27, 32, 37, 39, 40, 44, 47, 49, 52–59, 61, 67, 70, 72, 75, 77–79, 81, 83, 85, 87, 90, 93, 100, 102–104, 111–117]. Faculty from the schools of medicine, nursing, pharmacy and dentistry as well as faculty members from the departments of basic, clinical and allied sciences participated in these studies. The majority of outcomes reported were at level 2B (88.88%). The majority of faculty development programs led to significant increases in faculty members’ cognitive knowledge and skills of different aspects of assessment of students’ learning such as development of MCQs, giving effective feedback and workplace-based assessment.

#### The teacher as a role model

Five of faculty development programs focused on the domain of role model [70, 90, 93, 105, 118]. University-based and community-based general medicine faculty and as well as a mix of medical educators from multiple schools participated in these studies. The majority of outcomes reported were at level 2A (100%). Changes in attitudes and perceptions attributable to the educational program were infrequently noted.

#### The teacher as a manager and leader

Nineteen of faculty development programs focused on the domain of manager and leader [18, 23–25, 40, 49, 51–53, 58, 61, 65, 87, 90, 100, 106, 119–121]. Faculty from the colleges of medicine, dentistry, nursing and pharmacy and as well as basic science teachers from different departments and clinical teachers in family medicine, general pediatrics, and general internal medicine participated in these studies. The majority of outcomes reported were at level 2B (94.73%). The majority of faculty development programs led to significant increases in faculty members’ cognitive knowledge and skills of different aspects of the educational leadership and management such as time management, change management, small-group leadership, leadership and management of work teams, educational leadership and hospital administration and management. Participants also reported an improvement in their clinical leadership skills and financial skills.

#### The teacher as a scholar and researcher

Twenty nine of faculty development programs focused on the domain of scholar and researcher [14, 21, 24, 25, 27, 29, 47, 51–54, 58, 61, 80, 83, 84, 87, 100, 106, 112, 122–130]. Participants in these studies included faculty from the schools of medicine, dentistry, nursing and pharmacy and as well as entry level medical teachers and junior/mid-career clinical teachers in family medicine, general pediatrics, critical care and general internal medicine. The majority of outcomes reported were at level 2B (89.65%). The majority of faculty development programs led to significant improvement in participants’ self-reported knowledge and skills of different domains of the educational research and scholarship of teaching and learning, such as research design, critical appraisal of the literature, scientific writing, writing grant applications and evidence-based medicine (EBM). Participants also reported an improvement in their technology/informatics skills (e.g., search MEDLINE, use filters while searching, read journals on-line).

#### The teacher as a professional

Twenty four of faculty development programs focused on the domain of professional [16, 18, 25, 27, 29, 32, 34, 52, 54, 58, 61, 73, 76, 82, 83, 90, 100, 105–107, 118, 123, 129, 131]. A mix of professions, including medicine, nursing, pharmacy, dentistry, physical therapy, occupational therapy and speech-language pathology as well as junior/mid-career clinical faculty members and community-based physician faculty participated in these studies. The majority of outcomes reported were at level 2A (87.5%). Self-reported changes in attitudes included greater enthusiasm and motivation for lifelong learning, development or strengthening of responsibility for teaching, and an increased sense of community and collegiality. Participants also reported an increased understanding of individual professional development, career development, professional responsibility and interpersonal relationships.

(d) *Quality appraisal of the studies*

The quality of the studies was appraised using the Medical Education Research Study Quality Instrument (MERSQI). The overall methodological quality of studies was moderate. Total MERSQI scores amongst the 119 studies ranged from 7 to 16, with a mean (standard deviation) of 11.32 (0.74).

### Study design

The majority of studies (*n* = 72, 60.5%) used a single-group with a pre- and post- test design, with the addition of a delayed post-test in some programs. Forty three (36.13%) studies out of 119 were nonrandomized study with pre-test post-test design and there were four (3.36%) randomized controlled trials. Of the 119 articles reviewed, two authors employed a qualitative methodology only, though 19 (15.96%) researchers used a mixed methodology in their work.

### Sampling (institutions)

Although in most programs (*n* = 74, 62.18%), participants were from one institution, four studies were designed for the faculty of two institutions. In 41 studies, faculty from 3 or more institutions participated in the programs.

### Sampling (response rate)

In most programs (nearly 80% of programs), the response rate was ≥ 75%. Nearly 14.5% of studies (*n* = 17) had a response rate ranging from 50 to 74%. Seven studies reported a response rate of less than 50%.

### Type of data

More than half of the studies (51.26%) used participants’ self-report data to assess program outcomes. In 58 studies, in addition to self-reported data, objective data sources (e.g., expert opinion, student or resident ratings, student exam scores, retention rates, or success in promotion/tenure, engagement index, CV review) were also used.

### Validity evidence for evaluation instrument scores

Of the 119 articles reviewed, fifty eight (nearly 49% of the studies) did not measure a psychological construct. Forty three studies (nearly 36% of the reviewed publications) reported relevant content evidence included using theory, guidelines, experts, and existing instruments to identify or refine the instrument. In 11 studies, in addition to validity evidence for content, relevant internal structure evidence included all reliability (internal consistency, interrater, interstation, and test–retest) and factor analysis were reported. Moreover, seven studies reported relevant evidence of relationships to other variables included concurrent or predictive correlation with other variables.

### Data analysis (sophistication)

Although fifteen studies used descriptive analysis only, in 104 studies, in addition to descriptive analyses (e.g., frequency, mean, and median), statistical inference tests were also used.

### Data analysis (appropriate)

In most studies (nearly 93.5% of the reviewed articles), data analysis was appropriate for study design and type of data.

### Outcome

In previous section *(*section *b)*, we reviewed all included studies to determine which level of outcomes were assessed.

## Discussion

This review, which focused on faculty development programs designed to prepare faculty members for their various roles in health sciences education, included 119 articles published over a 30-year period. In this review, we searched the empirical health professions faculty development studies, both national and international, to determine the types and outcomes of faculty development programs designed to prepare faculty members for their various roles based on the Harden teacher’s role framework model and to identify, or explain, to which of the areas described by Harden and Crosby the authors are referring. In this section, first, we discussed the results of the review based on the research questions outlined in this paper. Next, we recommended several implications for future practice and research in the field. Finally, the authors presented the strengths and limitations of the review.

### Responses to review questions

In response to the first question of review, what are the types and duration of faculty development programs designed to prepare faculty members for their various roles in health sciences education, reviewers observed that the majority of faculty development programs were workshops with various durations, followed by longitudinal programs, short courses and seminar series. In considering the different approaches to faculty development illustrated by Steinert [132], it appears that the majority of faculty development programs in this review were conducted through formal approaches and occurred mostly in groups. This means that it has been less focused on the informal faculty development activities and as well as formal, individualized form of faculty development programs (e.g., peer coaching, peer & student feedback, and online learning). Although our review of findings by intervention type is consistent with previous literature reviews [6, 7, 9, 10, 133], it has recently been suggested that faculty developers incorporate more informal faculty development approaches (e.g., role modeling, reflection, and learning from peers) into the design and delivery of faculty development programs [134–136]. In one study [135], researchers identified three everyday educational practices (applying evidence to educational practice; and evaluating and sharing educational practice) that provide opportunities for informal faculty development for health professions educators in the academic setting. Providing informal learning opportunities in authentic contexts, can also lead to a community of practice [137]. As O’Sullivan and Irby [138] have noted, it would be timely to consider the role of workplace learning and communities of practice in investigating the effectiveness of faculty development programs and activities.

In our review, the duration of faculty development programs varied depending on the type of intervention and ranged from 20 min to six years. Interestingly, reviewers observed a notable shift in the length and format of faculty development programs (from one-time workshops to longitudinal programs such as teaching scholars programs, seminar series, fellowships and mentorship programs). As Gruppen [139] noted, intensive longitudinal programs are not only an investment in faculty; they are an investment in institutional health. The dynamic nature of aligning the goals of an intensive longitudinal program with the institution can promote educational leadership and scholarly productivity, and build a sense of community in the workplace.

In general, given the key features of effective faculty development (the role of experiential and authentic learning, the value of feedback and reflection, the importance of peers as role models and as providers of collegial support and the value of extended programs) highlighted in the literature [5, 7], it is time to reflect on the length and format of faculty development programs.

In response to the second question of review, which of the areas, or faculty^,^ roles, described by Harden and Crosby are the authors referring to, our findings showed that the majority of authors focused on the role of the teacher as an information provider and coach. Moreover, the domains of facilitator of learning and mentor and assessor and diagnostician are of interest for authors in the faculty development field. A much smaller number focused on the roles that faculty development programs can play in the preparation of faculty members for their roles as scholar and researcher and professional. Few articles focused on the roles of faculty members as manager and leader as well as curriculum developer and implementer. Only five faculty development programs emphasized on the role of the teacher as a role model. These findings mean that although we have seen a growth in the domains of faculty development programs, specifically in faculty development for research and scholarship, as well as leadership development, this growth has not been balanced and the majority of faculty development programs still tend to focus on enhancing the traditional role of faculty members as transmitters of information or lecturers. Clearly, focusing on the role of faculty members as role models merits further development and inquiry. Although reasons for this unbalanced growth may vary, including an emphasis on teaching effectiveness in teacher evaluation systems, focus on knowledge and skill acquisition rather than the formation – or development of – faculty members’ professional identity, overemphasis on faculty perceived or self-identified needs, institutional policies, as well as limited resources for faculty development programs, the need to expand the areas of faculty development programs remains acute. As highlighted by Steinert [2], it is time for us to broaden the scope of faculty development programs from teaching to academic development and intentionally design new programs targeting all of the roles that faculty members play. As implied at the outset, and as a number of authors have proposed [4, 140–143], utilizing a competency-based framework for faculty development (e.g. Harden teacher’s role framework) can play an important role in expanding our approaches to faculty development programs and would help to inform both content and desired outcomes [2]. Moving forward, it would be worthwhile to consider these frameworks in the design and evaluation of faculty development programs.

In response to the third question of review, we discussed the outcomes of faculty development programs based on the Harden teacher’s role framework described in Fig. 1 in Appendix 3 (see Additional file 4). Our findings showed that the majority of outcomes reported are at level 2B, level 1 and level 2A. This means that there were an overwhelming number of positive changes in participants’ attitudes, knowledge and skills in all eight domains of faculty roles following participation in faculty development programs, which suggests that faculty development programs were beneficial. The impact on the organizational practice as well as the impact on participants’ students, residents or colleagues continued to be underexplored, which may be disappointing. Outcomes of this review are consistent with Steinert^,^ study in 2016 [6], where the major outcome was teaching performance.

### Implications for future practice

Based on the review findings, we recommend the following suggestions for future practice:

-  *Utilizing a competency-based framework for developing a comprehensive faculty development program*
  Although we have seen a growth in the domains of faculty development programs, this growth has not been balanced and the majority of faculty development programs have been designed independently of a curriculum for faculty members, often in response to perceived or self-identified needs. Moving forward, we should adopt a competency-based framework (e.g. Harden teacher’s role framework) to program development, implementation and evaluation. As mentioned previously, utilizing a competency-based framework for faculty development can be considered as a guide for designing and evaluating faculty development programs and would help to address all faculty members’ roles.
- *Broadening the scope of faculty development programs beyond the traditional roles of the faculty members*
  As stated earlier, there has been a steady growth in the scope of faculty development programs and our findings showed that the majority of faculty development programs covered more than one domain of faculty roles. Nevertheless, most of these programs continued to focus on enhancing the traditional role of faculty members as information providers and assessors, often in an academic setting. Given the expanding number of roles of faculty in the health professions, there is a critical need to broaden the scope of faculty development programs outside of the academic milieu to target all health professions teachers’ roles.
- *Incorporating more informal approaches into the design and delivery of faculty development programs*
  The majority of faculty development programs in this review were conducted through formal approaches and occurred mostly in groups. Whereas these formal, structured approaches such as workshops, short courses, seminars and other longitudinal programs are expected and reflect the growth of the field to incorporate more systematic planning and program design [6], informal approaches to faculty development that take advantage of experiential learning in authentic environments, which include learning by doing, by observing, and by reflecting on experience as well as workplace learning and learning in a community of practice, should be considered.
- *Attention to individualized form of faculty development programs*
  As mentioned above, the majority of faculty development programs in this review took place primarily in groups (e.g. workshops of varying duration, short courses, seminar series and other longitudinal programs). With a nod to social learning theories and the role of feedback in promoting change, faculty developers can develop faculty expertise through individual approaches to faculty development such as learning from experience, learning from peers and students, peer coaching and online learning.
- *Moving fromworkshop-based teaching to workplace learning*
  In this review, faculty development programs were most commonly delivered through workshops that were not situated in the workplace, requiring participants to take their ‘lessons learned’ back to their own contexts. Given the challenges of using this approach for faculty development in the health professions in an ever-changing (and complex) environment, including integrating strategies to promote transfer to the workplace, this trend should be changed. Clearly, a re-orientation of activities from the workshop to the authentic environments such as workplace can increase participation, motivation, and access [2]. Moreover, by moving to the workplace and taking advantage of working together and participating in the activities of a larger community, faculty can build new knowledge and develop new approaches to challenges encountered in teaching and practice [144].
- *Fostering intensive longitudinal programs*
  We observed a notable shift in the length and format of faculty development programs (from one-time workshops to longitudinal programs such as teaching scholars programs, seminar series, fellowships and mentorship programs). Longitudinal programs that extend over time, have the potential to produce outcomes not apparent in one-time interventions. It is also not surprising that brief, one-time interventions are unlikely to have a significant impact on role modeling or reflective practice [134]. Given the multiple benefits of longitudinal approaches to faculty development noted in this review, other approaches that complement longitudinal programs included certificate programs and advanced degrees are needed.
- *Promoting role modeling and reflective practice*
  Although role modeling and reflective practice are important elements in all faculty roles [134], only 5% of faculty development programs in this review focused on the domain of role model. Faculty development for role modeling necessitates an awareness of the power of this teaching and learning strategy, attention to personal and professional behaviors, and a focus on the environment in which professional practice unfolds. Reflective practice is closely tied to role modeling and increased reflection enhances role modeling in all faculty roles [145]. Providing opportunities for feedback and reflection, which allowed participants to reflect on their practices, values, and beliefs, can promote role modelling.
- *Moving from focusing on knowledge and skill acquisition to the formation – or development of – faculty members’ professional identity*
  In this review, most faculty development programs focused on knowledge and skill acquisition rather than the formation – or support of – faculty members’ professional identity. As professional identity can have a powerful impact on academic and career development, academic roles and responsibilities, and professional development opportunities [146], we should strengthen identity through faculty development activities such as building opportunities for community building and networking; promoting reflection; and capitalizing on mentorship.
- *Moving from measurement of outcomes at the individual level to the organizational level*
  The current literature demonstrates a continuing overreliance on measurement of outcomes at the individual level (changes in cognitive learning or performance). As faculty development can play an important role in promoting organizational change and development, we need more programs to report on changes in organizational systems, as well as changes in student (or resident) behavior.

### Implications for future research

To build upon and complement existing research, we suggest the following recommendations for future faculty development studies:

- *Conducting more rigorous research designs, incorporating control or comparison groups or time series studies*
  As mentioned previously, the overall methodological quality of included studies was moderate and the majority of studies continued to employ a single-group with a pre- and post- test design. There is a need for more rigorous research designs and a greater use of control or comparison groups or time series studies to assess the impact of the faculty development intervention.
- *Make more deliberate use of qualitative methodologies and mixed methods approaches*
  Of the 119 articles reviewed, two authors employed a qualitative methodology only, though 19 researchers used a mixed methodology in their work. We need to conduct more qualitative and mixed methods studies to capture the complexity of faculty development interventions, as well as, to better understand the process of change, both as a result of the intervention and within the individual and the organization.
- *Using a more robust approach to data collection to assess program outcomes*
  This review demonstrates a continuing overreliance on participants’ self-report data and survey questionnaires to assess program outcomes. Moreover, nearly 49% of the studies did not measure psychometric properties. Moving forward, researchers should consider greater use of novel assessment methods for assessing behavioral and organizational changes. They also should use validated measures, or report validity evidence for evaluation instruments that are used. It would also be important to collect data over time, to better understand the long-term retention of outcomes.
- *Assessing change and the transfer of knowledge to practice over time*
  Although a number of studies used delayed post-tests for assessing the maintenance of change over time, there is a need for more studies to explore further the durability of change, those supports which help to sustain it, and the value of specific activities such as ‘booster’ sessions or other follow-up activities.
- *Exploring the impact of faculty development programs on the organizational development and change in a more rigorous and systematic fashion*
  As mentioned previously, most of the literature to date focuses on measurement of outcomes at the individual level (changes in cognitive learning or performance). To move forward, more studies is needed to observe the potential impact of faculty development programs on the organizational change. Such studies will help us to better understand the benefits of faculty development in producing organizational change.
- *Carry out process-oriented studies in addition to outcome-oriented ones*
  As we observed, most of the literature to date focuses on measurement of outcomes. There is a need for more qualitative studies to better understand how change occurs in faculty development, both as a result of the intervention and within the individual and the organization.
- *Grounding faculty development studies in theoretical foundation*
  Moving forward, we should adopt a sound theoretical basis for our research studies. We should also utilize theory in the interpretation of our results. From the perspective of external validity, it is critical that researchers base their studies on theoretical foundation.
- *Exploring the impact of faculty development programs in a single field of health sciences education*
  This systematic review included empirical health professions faculty development studies that assessed the impact of faculty development programs in all disciplines of health sciences. A more precise and dedicated analysis could have been the review and quantitative and qualitative synthesis of studies on only one of the disciplines of health sciences. This might be a future effort that can explicitly explore the impact of faculty development programs in a single field of health professions education.

### Strengths and limitations of the review

As noted in background, a major strength of this review is that our study is the first systematic review of faculty development programs designed to prepare faculty members for their various roles in health sciences education based on the Harden teachers’ role framework model. The use of a structured Coding Sheet, which was developed from previous systematic review work [7], was an additional strength. In addition, we appraised the quality of the studies using a reliable tool for appraising methodological quality of medical education research, the Medical Education Research Study Quality Instrument (MERSQI). Moreover, we used the modified Kirkpatrick model to assess the educational outcomes of faculty development programs. It is also noteworthy that the search process was extensive and in addition to international databases, national databases were also searched from 1990 to 2020.

There are some limitations to this study. A potential limitation of this study was the exclusion of program reports, dissertations and empirical studies with post-test design only from this review. Furthermore, articles not written in the English or Persian language were excluded. This means that this review may have been influenced by publication bias that prevents a fuller picture of faculty development from an international perspective. Negative results are also rarely reported, reflecting another possible publication bias towards positive results. It should also be noted that a complex search strategy in a field such as this one, where the terminology is still inconsistent across international and professional boundaries [7], created numerous challenges during the search process. Despite the rigorous process we undertook to achieve consensus, inter-rater reliability and agreement on the Coding Sheet was a challenge throughout the review process. Finally, the nature of the articles reviewed presented a number of challenges. For example, authors frequently omitted validity evidence for evaluation instruments that are used. In addition, an inconsistent use of terminology (e.g., to describe program types or study designs) often led to conflicting interpretations of the same information.

## Conclusion

In this review, we searched the empirical health professions faculty development studies published over a 30-year period, both national and international, to determine the types and outcomes of faculty development programs designed to prepare faculty members for their various roles based on the Harden teacher’s role framework model and to identify, or explain, to which of the areas described by Harden and Crosby the authors are referring. We observed a significant growth in faculty development programs world-wide. The majority of faculty development programs in this review were conducted through formal approaches and occurred mostly in groups (e.g., workshops with various durations, longitudinal programs, short courses and seminar series). Reviewers observed a notable shift in the length and format of faculty development programs (from one-time workshops to longitudinal programs such as teaching scholars programs, seminar series, fellowships and mentorship programs). Although we have seen a growth in the domains of faculty development programs, specifically in faculty development for research and scholarship, as well as leadership development, this growth has not been balanced and the majority of faculty development programs still tend to focus on enhancing the traditional role of faculty members as transmitters of information or lecturers. There were an overwhelming number of positive changes in participants’ attitudes, knowledge and skills in all eight domains of faculty roles following participation in faculty development programs, which suggests that faculty development programs were beneficial. The impact on the organizational practice as well as the impact on participants’ students, residents or colleagues continued to be underexplored, which may be disappointing. Lastly, based on the review findings, we recommended several implications for future practice and research in the field.

### Supplementary Information

**Additional file 1:** **Appendix 1.** A copy of the search strategy used within MEDLINE/PubMed database.

**Additional file 2:** **Appendix 2.** The Coding Sheet.

**Additional file 3:** **Table S1. **Summary of included studies.

**Additional file 4:** **Appendix 3.** The study findings based on the Harden teacher’s role framework.

## Data Availability

All data generated or analysed during this review are included in this published article and its additional files.

## Abbreviations

BEME: Best Evidence Medical Education
CV: Curriculum Vitae
EBM: Evidence-based Medicine
ED: Emergency Department
ERIC: Education Resources Information Center
IPE: Interprofessional Education
MCQs: Multiple-Choice Questions
MERSQI: Medical Education Research Study Quality Instrument
mini-CEX: Mini-Clinical Evaluation Exercise
OSTE: Objective Structured Teaching Exercise
PBL: Problem-Based Learning
Ph.D.: Doctor of Philosophy
PRISMA: Preferred Reporting Items for Systematic Reviews and Meta-Analyses
QI: Quality Improvement
RN/MD: Registered Nurse/ Medical Doctor
SID: Scientific Information Database
