# Supplementary material for: A systematic review of faculty development programs based on the Harden teacher’s role framework model
Source: BMC Med Educ. 2023 Nov 30;23:910. doi: 10.1186/s12909-023-04863-4 (PMC10690997; doi:10.1186/s12909-023-04863-4)
Supplement: Supplementary file 1 — Additional file 1: Appendix 1. A copy of the search strategy used within MEDLINE/PubMed database. [file 12909_2023_4863_MOESM1_ESM.pdf]

Appendix 1. A copy of the search strategy used within MEDLINE/PubMed database.

((((((((((((((((("faculty development" AND free full text[sb] AND ("1990/01/01"[PDat] : "2019/12/31"[PDat]) AND Humans[Mesh] AND English[lang])) OR ("faculty empowerment" AND free full text[sb] AND ("1990/01/01"[PDat] : "2019/12/31"[PDat]) AND Humans[Mesh] AND English[lang])) OR ("Faculty training" AND free full text[sb] AND ("1990/01/01"[PDat] : "2019/12/31"[PDat]) AND Humans[Mesh] AND English[lang])) OR ("faculty education" AND free full text[sb] AND ("1990/01/01"[PDat] : "2019/12/31"[PDat]) AND Humans[Mesh] AND English[lang])) OR ("teacher education" AND free full text[sb] AND ("1990/01/01"[PDat] : "2019/12/31"[PDat]) AND Humans[Mesh] AND English[lang])) OR ("teacher training" AND free full text[sb] AND ("1990/01/01"[PDat] : "2019/12/31"[PDat]) AND Humans[Mesh] AND English[lang])) OR ("teacher improvement" AND free full text[sb] AND ("1990/01/01"[PDat] : "2019/12/31"[PDat]) AND Humans[Mesh] AND English[lang])) OR ("teacher promotion" AND free full text[sb] AND ("1990/01/01"[PDat] : "2019/12/31"[PDat]) AND Humans[Mesh] AND English[lang])) OR ("faculty growth" AND free full text[sb] AND ("1990/01/01"[PDat] : "2019/12/31"[PDat]) AND Humans[Mesh] AND English[lang])) OR ("faculty improvement" AND free full text[sb] AND ("1990/01/01"[PDat] : "2019/12/31"[PDat]) AND Humans[Mesh] AND English[lang])) OR ("faculty promotion" AND free full text[sb] AND ("1990/01/01"[PDat] : "2019/12/31"[PDat]) AND Humans[Mesh] AND English[lang])) OR ("inservice teacher education" AND free full text[sb] AND ("1990/01/01"[PDat] : "2019/12/31"[PDat]) AND Humans[Mesh] AND English[lang])) OR ("professional development" AND free full text[sb] AND ("1990/01/01"[PDat] : "2019/12/31"[PDat]) AND Humans[Mesh] AND English[lang])) AND free full text[sb] AND ("1990/01/01"[PDat] : "2019/12/31"[PDat]) AND Humans[Mesh] AND English[lang])) AND (((((((((((model AND free full text[sb] AND ("1990/01/01"[PDat] : "2019/12/31"[PDat]) AND Humans[Mesh] AND English[lang])) OR (system AND free full text[sb] AND ("1990/01/01"[PDat] : "2019/12/31"[PDat]) AND Humans[Mesh] AND English[lang])) OR (program AND free full text[sb] AND ("1990/01/01"[PDat] : "2019/12/31"[PDat]) AND Humans[Mesh] AND English[lang])) OR (center AND free full text[sb] AND ("1990/01/01"[PDat] : "2019/12/31"[PDat]) AND Humans[Mesh] AND English[lang])) OR (framework AND free full text[sb] AND ("1990/01/01"[PDat] : "2019/12/31"[PDat]) AND Humans[Mesh] AND English[lang])) OR (process AND free full text[sb] AND ("1990/01/01"[PDat] : "2019/12/31"[PDat]) AND Humans[Mesh] AND English[lang])) OR (Structure AND free full text[sb] AND ("1990/01/01"[PDat] : "2019/12/31"[PDat]) AND Humans[Mesh] AND English[lang])) OR (activity AND free full text[sb] AND ("1990/01/01"[PDat] : "2019/12/31"[PDat]) AND Humans[Mesh] AND English[lang])) OR (component AND free full text[sb] AND ("1990/01/01"[PDat] : "2019/12/31"[PDat]) AND Humans[Mesh] AND English[lang])) AND free full text[sb] AND ("1990/01/01"[PDat] : "2019/12/31"[PDat]) AND Humans[Mesh] AND English[lang])) AND (((((((((((("medical education" AND free full text[sb] AND ("1990/01/01"[PDat] : "2019/12/31"[PDat]) AND Humans[Mesh] AND English[lang])) OR ("program evaluation" AND free full text[sb] AND ("1990/01/01"[PDat] : "2019/12/31"[PDat]) AND Humans[Mesh] AND English[lang])) OR ("medical faculty" AND free full text[sb] AND ("1990/01/01"[PDat] : "2019/12/31"[PDat]) AND Humans[Mesh] AND English[lang])) OR ("medical teacher" AND free full text[sb] AND ("1990/01/01"[PDat] : "2019/12/31"[PDat]) AND Humans[Mesh] AND English[lang])) OR ("clinical teacher" AND free full text[sb] AND ("1990/01/01"[PDat] : "2019/12/31"[PDat]) AND Humans[Mesh] AND English[lang])) OR ("health

sciences education" AND free full text[sb] AND ("1990/01/01"[PDat] : "2019/12/31"[PDat]) AND Humans[Mesh] AND English[lang])) OR ("health sciences educator" AND free full text[sb] AND ("1990/01/01"[PDat] : "2019/12/31"[PDat]) AND Humans[Mesh] AND English[lang])) OR ("pharmacy faculty" AND free full text[sb] AND ("1990/01/01"[PDat] : "2019/12/31"[PDat]) AND Humans[Mesh] AND English[lang])) OR ("nursing faculty" AND free full text[sb] AND ("1990/01/01"[PDat] : "2019/12/31"[PDat]) AND Humans[Mesh] AND English[lang])) OR ("dental faculty" AND free full text[sb] AND ("1990/01/01"[PDat] : "2019/12/31"[PDat]) AND Humans[Mesh] AND English[lang])) AND free full text[sb] AND ("1990/01/01"[PDat] : "2019/12/31"[PDat]) AND Humans[Mesh] AND English[lang])
