# Supplementary material for: A systematic review of faculty development programs based on the Harden teacher’s role framework model
Source: BMC Med Educ. 2023 Nov 30;23:910. doi: 10.1186/s12909-023-04863-4 (PMC10690997; doi:10.1186/s12909-023-04863-4)
Supplement: Supplementary file 2 — Additional file 2: Appendix 2. The Coding Sheet. [file 12909_2023_4863_MOESM2_ESM.pdf]

Appendix 2. The Coding Sheet.

Authors (year)	Country and institution	Type of Program or Intervention and duration	Participants (n)	Faculty' roles covered by the program (The teacher as :)	Study design	Outcomes	Outcome Level						Study quality. MERSQI score out of 18
							1	2A	2B	3	4A	4B	
