# Supplementary material for: A systematic review of faculty development programs based on the Harden teacher’s role framework model
Source: BMC Med Educ. 2023 Nov 30;23:910. doi: 10.1186/s12909-023-04863-4 (PMC10690997; doi:10.1186/s12909-023-04863-4)
Supplement: Supplementary file 3 — Additional file 3: Table S1. Summary of included studies. [file 12909_2023_4863_MOESM3_ESM.pdf]

Table S1 Summary of included studies.

Authors (year)	Country and institution	Type of Program or Intervention and duration	Participants (n)	Faculty' roles covered by the program (The teacher as :)	Study design	Outcomes	Outcome Level						Study quality. MERSQI score out of 18
							1	2A	2B	3	4A	4B	
Stein et al. (2012)	USA, Washington State University	A 1-day teaching-methods course	12 pharmacy and nursing faculty members	Information provider and coach	One group, Pretest-posttest	Significant improvement in 7 of 10 domains of teaching effectiveness; self-reported changes in participants' perceptions of their teaching effectiveness.		√	√				13
Zagar et al. (2019)	USA, University of Louisiana at Monroe	A year-long book club	48 pharmacy faculty members	Information provider and coach-Facilitator of learning and mentor-Assessor and diagnostician - Scholar and researcher	One group, Pretest-posttest	Increased ability to describe the characteristics of a student-centered approach to teaching, the components of the pharmacy education system, the components of assessment in	√	√	√	√			9.5

						pharmacy education and the roles and responsibilities of a student mentor.							
Fleming et al. (2015)	USA, Vanderbilt University School of Medicine	A facilitated peer mentoring program, monthly for 1.5 hours for 2 years	104 junior faculty in the Department of Pediatrics	Professional-Scholar and researcher	Cohort with a survey at two time points	Significant improvement in participants' self-reported knowledge, skills, and attitudes (KSA) in the domains of professional development and scholarship, including the ability to write career goals and align activities with those goals.		√	√				10.5
Grimes et al. (2000)	USA, Berlex Foundation	A 6-day faculty development course	65 faculty physicians	Scholar and researcher	One group, Pretest-posttest	Increases in participants' cognitive knowledge of research			√				10
Johnstone-	USA, forty-	A four-week online	5 dental	Information provider and	One group,	Significant increases in	√	√					11.5

Dodge et al. (2014)	seven U.S. dental hygiene programs	faculty development course	hygiene faculty members	coach-Facilitator of learning and mentor	Pretest-posttest	participants' perceptions of the importance of four practices: activities promoting relevant, lifelong learning; faculty communication fostering a sense of community; encouraging students' self-introduction; and encouraging productive dialogue and respecting diverse opinions.							
Jackevicius et al. (2014)	USA, Western University of Health Sciences	A four-year formal mentorship program	51 pharmacy faculty mentor-protégé´ pairs	Scholar and researcher	One group, Pretest-posttest	Non-significant improvements in the protégés' number of grant submissions,	√			√			11

						retention rates, or success in promotion/tenure; significant increases in the total number of peer-reviewed publications for junior faculty protégés.							
Batsche & Monoson (1993)	USA, Illinois State University	A 17-month four-phase multidisciplinary faculty development	10 faculty members in the areas of social services, human performance, psychology, nutrition, audiology, and nursing.	Information provider and coach-Curriculum developer and implementer-Scholar and researcher	Non-equivalent control group, Pretest-posttest	Significant increases in faculty knowledge in the field of aging; significant gains in students' knowledge in gerontological concepts; 10 faculty members developed 50 instructional Units.			√	√	√	√	13.5
Taylor &	USA, St. Louis	A year-long Pharmacy	21 new	Professional	One group,	More highly engaged and	√			√			11.5

Berry (2008)	College of Pharmacy	Faculty Academy	pharmacy faculty members		Pretest-posttest	productive faculty members who were more likely to remain long term within the college.						
Johansson et al. (2012)	Sweden, Uppsala University Hospital	14-hour Stanford faculty development programme over a 7-week period	48 physicians from different departments	Information provider and coach	Randomised controlled study, Pretest-posttest	Positive changes in teaching behaviors as assessed by the participants' self-ratings.	√			√		12
Windish et al. (2007)	USA, Johns Hopkins Bayview Medical Center	A 10-month longitudinal mentored faculty development program	138 faculty and fellows from Johns Hopkins and other institutions and 63 matched nonparticipants	Curriculum developer and implementer	Non-equivalent control group, Pretest-posttest	Improvement in self-assessed curricular development, implementation, and evaluation skills and enjoyment of participants.	√			√		14
Lee et al. (2012)	USA, University of California	A 4-month Web-based train-the-trainer program	58 pharmacy faculty trainers	Information provider and coach	One group, Pretest-posttest	Significant increases in instructors' self-reported ability to teach	√	√		√		11.5

						pharmacogenomics to pharmacy students							
Green (2005)	USA, seven podiatric medical colleges	A 2-day workshop	10 academic podiatric physicians	Information provider and coach-Curriculum developer and implementer-Scholar and researcher	One group, Pretest-posttest, delayed post-test	Improvement in participants' self-reported EBM practice and EBM teaching skills and, within 3 months, fully or partially implemented many changes in their clinical practice, podiatric medical teaching, and administrative duties.		√	√	√	√		12.5
Smith et al. (2018)	USA, Long Island Jewish Forest Hills	Three OSTE simulation sessions lasting about 2.5 h over a 2-month period	29 attending physicians	Information provider and coach	One group, Pretest-posttest, delayed post-test	Non-significant increases in 'intern' OSTE ratings of attendings and attending self-assessment ratings and			√				13

						resident evaluations of attendings.							
Gjerde et al. (2004)	USA, University of Wisconsin	A year-long series of five weekend workshops over 6 years (A six-year faculty development fellowship program)	84 clinical teachers in family medicine, general pediatrics, and general internal medicine	Information provider and coach- Manager and leader- Scholar and researcher	One group, Pretest-posttest	Significant improvement in participants' self-reported EBM skills, teaching skills, technology/ informatics skills and leadership/advocacy skills.	√		√				10
Sabato et al. (2017)	USA, Rutgers School of Dental Medicine	The two-year program with weekly meetings	35 dental faculty members	Information provider and coach- Manager and leader	4 cohort, Pretest-posttest	Significant increases in participants' perception of preparedness in administrative, clinical, and educational competencies.	√		√				9.5
LeBlanc et al. (2007)	USA, Ohio State University College of Pharmacy	A 2-part training seminar includes 42 credit hours of online	22 pharmacy instructors	Information provider and coach- Facilitator of learning and mentor	One group, Pretest-posttest	Significant increases in level of comfort in their ability to facilitate online case-	√	√					9.5

		didactic course work				based discussions.							
Manwell et al. (2006)	Six universities throughout the United States	Two 2-day skills-based courses held 6 months apart	153 faculty from multiple disciplines at 6 locations	Information provider and coach-Scholar and researcher	One group, Pretest-posttest, delayed post-test	Significant improvements in the teaching, clinical, and research activities of faculty; increases in number of professional activities in the alcohol area; significant improvements in screening, brief intervention, and motivational interviewing skills.	√		√	√			12.5
Koffel & Reidt (2015)	USA, University of Minnesota	The two-day interprofessional evidence-based practice workshop	25 faculty from the schools and colleges of Medicine, Nursing, Pharmacy,	Information provider and coach-Scholar and researcher	One group, Pretest-posttest, delayed post-test	Significant improvements in faculty members' self-reported confidence and ability to practice and	√		√				10.5

			Dentistry, and Veterinary Medicine and the Center for Allied Health Programs			teach core EBP skills.							
Sturpe et al. (2013)	USA, University of Maryland School of Pharmacy	A two-station OSTE	14 faculty/residents	Information provider and coach	One group, Pretest-posttest	Most participants scored well on the standardized checklist items; all participants agreed the debriefing session was useful.	√		√				11
Brown et al. (2016)	Canada, University of Calgary	The four three-hour orientations to wellness workshop	47 new faculty of medicine members	Scholar and researcher-Professional	One group, Pretest-posttest	Significant increases in post workshop self-efficacy scores compared to pre workshop ratings; most participants expressed intention to change their	√	√	√	√			9.5

						behavior as a result of participating in the workshop.							
Roberts & Devries (2004)	USA, Institutions with community -based teaching programs	A series of three 2-day ‘‘Train the Trainer’’ workshops was spread over 12 months	42 academicians and practitioners	Information provider and coach- Facilitator of learning and mentor- Assessor and diagnostician - Manager and leader	2 cohort, Pretest-posttest	Significant improvement in participants’ self-reported knowledge, skills, and attitudes to recruit and retain community-based preceptors and increases in multiple areas of competence.	√	√	√	√	√		12
Ebrahimi & Kojuri (2012)	Iran, Shiraz University of Medical Sciences	A series of faculty empowerment workshops as one month medical education programs, four times per academic	219 basic science and clinical science teachers	Information provider and coach- Facilitator of learning and mentor- Assessor and diagnostician - Manager and leader	Equivalent control group, Pretest-posttest, delayed post-test	Significant positive effect on medical teachers’ competencies; significant improvements in the participants’ teaching abilities.	√	√	√	√			12

		year											
Shealy et al. (2019)	USA, University of South Carolina College of Pharmacy	The two-hour training and one-hour active-learning sessions of Birkman Method Faculty and Preceptor Development Intervention	38 pharmacy faculty members and 51 preceptors	Information provider and coach-Professional	One group, Pretest-posttest, delayed post-test	Significant improvement in participants' self-reported self-awareness and self-confidence related to coaching; significant changes in self-perception in usual communication style and behavior style under stress.		√	√	√			12.5
Willett (2006)	USA, UMDNJ-Robert Wood Johnson Medical School	The 20-min faculty development audiotape	53 academic and community preceptors	Information provider and coach	One group, Pretest-posttest, delayed post-test	Improvement in participants' teaching technique; change in shadowing behavior of preceptors.				√			11.5
Yanamadala et al. (2016)	USA, Duke University School of	6-month-long quality improvement (QI) course	8 faculty members from the	Manager and leader	One group, Pretest-posttest	Significant changes in participants' QI knowledge	√	√	√	√			13.5

	Medicine		Department of Medicine			and self-assessed confidence on key QI concepts.							
Johansson et al. (2009)	Sweden, Uppsala University Hospital	Five series of 7-week faculty development seminar	40 physicians from different departments	Information provider and coach	One group, Pretest-posttest	Significant increases in participants' teaching skills; positive changes in their teaching behaviours.	√	√	√	√			9.5
Lye et al. (1998)	USA, Medical College of Wisconsin	Seven 1-hour conferences (A Case-Oriented Faculty Development Program)	101 pediatric faculty attending	Information provider and coach-Facilitator of learning and mentor	Non-equivalent control group, Pretest-posttest	Significant improvement in both feedback and overall teaching effectiveness of faculty attending clinical teaching rounds.			√				11.5
Lang et al. (2012)	USA, University of Rochester School of Medicine and Dentistry	Monthly 1-hour interactive workshop on collaborative development of teaching scripts	22 internal medicine, or combined internal medicine and pediatrics	Information provider and coach-Professional	One group, Pretest-posttest, qualitative content analysis	No significant differences in quantity or applicability of teaching; significant improvement in faculty self-	√	√		√			10.5

						efficacy with teaching.							
Tenzin et al. (2017)	Bhutan, Khesar Gyalpo University of Medical Sciences of Bhutan	A 2 hours long workshop on construction of MCQs	16 intern medical doctors	Assessor and diagnostician	Quasi-experimental study	Effective changes in the learning and performances of medical educators in the development of MCQs.			√				12.5
McAndrew et al. (2012)	USA, New York University College of Dentistry	Three-station OSTE	12 faculty members	Information provider and coach	2 cohort, Pretest-posttest	Significant changes in faculty participants' OSTE scores and ratings; significant improvements in nine of the fifteen teaching domains.	√		√				12.5
Wong & Agisheva (2004)	Russia, Kazan State Medical University	Four seminars based on the 7 categories of the Stanford Faculty Development Program model	14 medical teachers	Information provider and coach	One group, Pretest-posttest, delayed post-test	Significant improvements in the ratings of global teaching performance and specific teaching behaviors at both measured	√		√				10

						times.							
Wong et al. (2017)	Japan, University of Tokyo Graduate School of Medicine	Two complete series of the seven 2-hour seminars was spread over 4 months	19 nurse and physician educators	Information provider and coach	One group, Pretest-posttest, delayed post-test	Significant improvements in participants' self-reported teaching abilities at both the posttest and at 1 year.			√				10
Paige et al. (2015)	USA, Association for Surgical Education	A train the trainer workshop with 2 separate 90-minutes sessions	----	Information provider and coach	One group, Pretest-posttest, delayed post-test	Significant improvements in participants' self-efficacy in objectives-driven aspects of debriefing.	√	√					9.5
Sullivan et al. (2006)	USA, Harvard Medical School	Two-week program offered annually (2000 to 2003) with 2 on-site sessions and 6-month distance-learning period	156 physician and nurse educators from North America and Europe	Information provider and coach- Facilitator of learning and mentor- Professional	4 cohort, Pretest-posttest, delayed post-test	Increases in: time spent in palliative care practice, use of learner-centered teaching approaches and palliative care topics taught; improvements in participants'	√	√	√	√	√		13.5

						self-reported clinical practices in psychosocial dimensions of care; major improvements in confidence, commitment to palliative care, and enthusiasm for teaching.							
Sigalet et al. (2016)	Qatar, Sidra Medical and Research Center	A two day interprofessional simulation based faculty development course	41 clinicians	Information provider and coach-Facilitator of learning and mentor-Professional	One group, Pretest-posttest	Significant improvements in aggregate MCQ scores from pre- to post-test; non-significant improvements in participants' ability to apply the theory of feedback.			√				12
Rao et al. (2017)	USA, Partners HealthCare	A 6-day interdisciplinary, team-based quality improvement leadership	13 cohorts consisting of a total of 239 teams and 516	Manager and leader	13 cohorts, Pretest-posttest, delayed post-test	Improvements in participants' self-reported understanding of QI	√	√	√	√	√		11

		training program over a period of 4 months	physicians, registered nurses, other clinicians and administrators			concepts.							
Roos et al. (2014)	Germany, Heidelberg Medical Faculty	A 5-day teaching education program	56 medical educators	Information provider and coach- Facilitator of learning and mentor- Assessor and diagnostician - Manager and leader- Role model- Professional	One group, Pretest-posttest, delayed post-test	Significant increases in participants' knowledge; improvements in participants' teaching performance ratings, transfer of learning into teaching performance by the participants.	√	√	√	√	√		12.5
Wong & Agisheva (2007)	Russia, Kazan State Medical University	Five small-group seminars based on the 7 categories of the Stanford Faculty Development Program	48 faculty members from 17 different academic departments	Information provider and coach	One group, Pretest-posttest, delayed post-test	Significant improvements in the ratings of global teaching performance and specific teaching behaviors at both measured	√		√	√			10.5

		model				times.						
Liao et al. (2013)	Taiwan, Chang Gung University	A two-hour mini-CEX workshop	49 trainers of different subspecialties	Facilitator of learning and mentor-Assessor and diagnostician	One group, Pretest-posttest	Significant improvements in participants' cognitive knowledge; enhancement of giving feedback to residents.	√		√	√			11.5
Arnett et al. (2017)	USA, University of Michigan School of Dentistry	A five-hour workshop	16 dental hygiene faculty members	Information provider and coach-Facilitator of learning and mentor	One group, Pretest-posttest, delayed post-test	Increases in dental hygiene faculty members' appreciation of the value of motivational interviewing and confidence in teaching it to students immediately after the workshop.		√	√				12
Register et al. (2019)	USA, University of Alabama at Birmingham	A five-hour standard and intensive interprofessional faculty development course in	51 faculty from multiple schools	Role model-Professional	Mixed methodology, Non-equivalent control	Better retention of positive interprofessional perceptions in the intensive		√					10

		simulation			group, Pretest-posttest, delayed post-test	training group; gradual decline in positive interprofessional perceptions in all groups.							
Baral et al. (2015)	Nepal, B.P. Koirala Institute of Health Sciences	2 two and a half day teacher-training workshops	31 faculty members from the departments of basic, clinical and allied sciences	Information provider and coach-Facilitator of learning and mentor-Assessor and diagnostician	One group, Pretest-posttest	Improvement in participants' knowledge on different aspects of medical education.	√	√	√				10
John et al. (2016)	India, Medical College in Kochi	Eight-hour training curriculum in teaching methodology	30 entry level medical teachers	Scholar and researcher	Quasi-experimental study with pre-test post-test design	Significant improvements in participants' scores for knowledge, motivation to teach and practice.	√	√	√				9.5
Mokkapati & Mada (2018)	India, Apollo Institute of Medical Sciences	A two day teacher training workshop	29 faculty members	Information provider and coach-Facilitator of learning and mentor-	One group, Pretest-posttest	Significant improvements in knowledge of the participants after the			√				11

	and Research			Assessor and diagnostician		workshop.							
Popovich et al. (2006)	USA, University of Illinois College of Pharmacy	Weekly 60–90-minute conversation forums	11 pharmacy faculty members	Information provider and coach-Facilitator of learning and mentor-Assessor and diagnostician - Professional	One group, Pretest-posttest	Significant self-perceived improvement from pretest to posttest.	√	√	√				10
Griffeth & Wiederman (2018)	USA, The University of South Carolina School of Medicine Greenville	60 minutes in total, divided over two training sessions embedded within the monthly hour-long faculty meeting.	33 faculty members	Assessor and diagnostician	One group, Pretest-posttest	Statistically significant differences in both the self-rated confidence of faculty members in their ability to accurately rate student performance and in the actual clinical assessment ratings of students after the intervention.	√	√	√	√			11.5
Sánchez -	Mexico, UNAM	A monthly one-hour	22 participants,	Assessor and diagnostician	Quasi-experi	Improvement in self-	√	√	√	√			11

Mendiola et al. (2015)	Faculty of Medicine in Mexico City.	medical education Journal Club	a mix of clinicians, educators, psychologists and one sociologist	- Scholar and researcher	mental study with pre-test post-test design	reported competencies in medical education literature critical appraisal and behaviors related to the use of evidence in educational practice.							
Haines & Popovich (2014)	USA, the Palm Beach Atlantic University Gregory School of Pharmacy	A nontraditional external faculty mentor program (monthly sessions and 2 annual summer retreats)	13 junior/mid-career clinical faculty members	Information provider and coach-Facilitator of learning and mentor-Scholar and researcher-Professional	One group, Pretest-posttest	Improvements in meeting faculty needs related to professional development, individual feedback, career development.	√	√	√				9
Sherbino et al. (2006)	Canada, University of Toronto	A novel one-day evidence-based, skills-oriented faculty development course	31 individuals from a variety of academic and community EDs	Information provider and coach	Mixed methodology, One group, Pretest-posttest, delayed post-	Improvements in participants' knowledge about ED-specific teaching strategies, and this	√	√	√	√			13.5

					test	improvement is maintained at one month.							
Mitcham et al. (2002)	USA, Medical University of South Carolina	10 occupational therapy faculty development workshops	179 occupational therapy faculty participants	Information provider and coach- Facilitator of learning and mentor- Assessor and diagnostician - Scholar and researcher- Professional	10 cohorts, Pretest-posttest	Changes in participants' current teaching practices.	√	√		√			8.5
Bigby & Barnes (1993)	USA, Brigham and Women's Hospital	A learner-centered, largely experiential faculty development program in substance abuse education	87 general internal medicine faculty from academic institutions	Information provider and coach	One group, Pretest-posttest	Changes in participants' knowledge, attitudes, and confidence in clinical skills.		√	√	√			12.5
Skeff et al. (1992)	USA, Stanford University School of Medicine	One-month faculty development program based on the 7 categories of the Stanford Faculty	29 clinical faculty members	Information provider and coach- Facilitator of learning and mentor- Assessor and diagnostician - Manager	One group, Pretest-posttest	Significant changes in participants' teaching performance and attitudes.	√	√	√	√			11.5

		Development Program model		and leader									
Assemi et al. (2007)	USA, schools across the United States	A 2-day cultural competence train-the-trainer workshop	50 pharmacy educators	Information provider and coach	One group, Pretest-posttest, delayed post-test	Significant increases in faculty members' perceived and documented ability to teach cultural competence.	√	√	√	√			10.5
Quirk et al. (1998)	USA, Different regions of United States	Five national series of faculty development workshops	223 community health center preceptors from a variety of disciplines	Information provider and coach-Assessor and diagnostician	One group, Pretest-posttest, delayed post-test	Significant increases in the teaching knowledge and skills and use of effective teaching behaviors.	√	√	√	√			12.5
Lye et al. (2003)	USA, Medical College of Wisconsin	A monthly faculty development program	7 experienced clinical educators	Information provider and coach	Non-equivalent control group, Pretest-posttest, delayed post-test	Significant increases in participants' use of 2 teaching methods (priming and feedback).			√	√			12
Baker et al. (2010)	Canada, University of	Five 1-day face-to-face sessions	32 participants, a mix of	Facilitator of learning and mentor-	One group, Pretest-	Significant improvements in the areas of		√	√				9

	Toronto	spread over three months (IPE faculty development program)	professions, including medicine, nursing, pharmacy, physical therapy, speech-language pathology and social work	Professional	posttest	knowledge, skills, community, and feelings of success and confidence about the principles of IPE and faculty development.							
Moore & Pinsky (2015)	USA, University of Pittsburgh	22 broad-based career development course sessions	12 critical care faculty and 1 non–critical care faculty	Curriculum developer and implementer-Facilitator of learning and mentor-Scholar and researcher-Professional-Manager and leader	One group, Pretest-posttest, delayed post-test	General increase in comfort scores between precourse and postcourse surveys and the maintenance of these scores for set of administrative, educational, research, and other professional development skills at 3-year follow-up.	√	√	√				7
Mookherjee et	USA, University	A year-long peer	22 attending	Information provider and	One group,	Significant increases in	√	√	√	√			10

al. (2014)	of California	observation, assessment, and feedback program	hospitalists	coach-Facilitator of learning and mentor-Assessor and diagnostician	Pretest-posttest	hospitalist confidence in giving feedback, receiving feedback, and teaching efficacy.							
Gardner et al. (2019)	USA, University of Texas Southwestern Medical Center	A 4-hour intraoperative teaching course	19 faculty members from the general surgery department	Information provider and coach-Facilitator of learning and mentor-Assessor and diagnostician	One group, Pretest-posttest, delayed post-test	Resident-perceived improvement in structured teaching behavior among participating faculty.	√			√			11.5
Gates et al. (2013)	USA, Bronx-Lebanon Hospital Center	A four-phase program comprising twelve days of seminars (three days per phase at seven contact hours per day)	15 dental education faculty members	Information provider and coach-Facilitator of learning and mentor-Assessor and diagnostician - Curriculum developer and implementer - Scholar and researcher-Professional-Manager and	One group, Pretest-posttest	Significant changes in participants' knowledge and confidence for a wide range of academic skills in the realms of learning theory, teaching strategies, curriculum design,	√	√	√				12

				leader		assessment of students' learning, educational leadership, scholarship, and career development.							
Wong et al. (2004)	USA, American Society of Addiction Medicine	A 1-day faculty development workshop	26 faculty small group facilitators	Information provider and coach- Facilitator of learning and mentor	One group, Pretest-posttest	Significant improvements in participants' small-group teaching skills.	√		√				10
Cole et al. (2004)	USA, Johns Hopkins University, School of Medicine	An intensive (3.5 hours weekly), longitudinal (nine months) faculty development program in teaching skills	228 Clinician–Educators (100 program participants and 128 program nonparticipants)	Information provider and coach- Facilitator of learning and mentor- Assessor and diagnostician - Professional- Manager and leader	Non-equivalent control group, Pretest-posttest	Significant changes in participants' self-assessed teaching and professional skills, teaching enjoyment, and learning effectiveness.	√	√	√	√			11
Salerno et al. (2002)	USA, Ambulatory internal medicine clinic in a tertiary	Three 90-minute faculty development seminars based on the	9 board-certified internist faculty preceptors	Information provider and coach- Facilitator of learning and mentor-	Mixed methodology, One group,	Significant improvements in the quality of verbal feedback delivered in	√		√	√			12.5

	care medical center	One-Minute Preceptor teaching model	and 44 third-year medical students	Assessor and diagnostician	Pretest-posttest	the ambulatory setting.							
Christie et al. (2007)	USA, Idaho State University	A four-hour faculty development workshop	14 clinical faculty members	Information provider and coach-Assessor and diagnostician	Mixed methodology, Three cohorts, Pretest-posttest, delayed post-test	The clinical faculty's evaluation of professional judgment during patient care was enhanced by training; written comments more frequently related to core values defined in the ADHA code of ethics; students were more positive in outcomes assessments about their competency and learning experiences related to professionalism and ethics.	√	√	√	√	√	√	13
Delver	Canada,	The 90-min	13	Information	Mixed	Significant	√	√					13

et al. (2014)	University of Calgary	Family Medicine Preceptor Online Development program	family medicine preceptors	provider and coach- Facilitator of learning and mentor	metho dology , One group, Pretest-posttest	increases in mean comfort with all the precepting skills taught: giving effective feedback, using questions to teach, teaching communications skills, helping learners in difficulty, and making teaching time-efficient.							
Srivastava et al. (2016)	India, Jawaharlal Nehru Medical College (JNMC)	A one-year Faculty Development Fellowship Program in Medical Education	40 faculty members from different medical schools	Scholar and researcher	Mixed metho dology , One group, Pretest-posttest	Changes in participants' knowledge regarding educational research.			√				9
Pololi & Frankel (2005)	USA, East Carolina University School of Medicine	A year-long faculty development programme	11 clinical faculty from 6 different clinical	Information provider and coach- Facilitator of learning and mentor	Qualitative themat ic and content analysi	Significant improvements in participants' self-awareness,	√	√	√				9

			departments		s	teaching skills and habits of lifelong learning.							
Lee et al. (2018)	USA, University of Chicago	The 4-hour faculty workshop consisted of a lecture and a Group-Objective Structured Clinical Exam (GOSCE)	30 Family Medicine and General Internal Medicine Faculty	Information provider and coach-Facilitator of learning and mentor	Randomised controlled study, Pretest-posttest, delayed post-test	No differences were found between longer versus shorter training; improvements in participants' patient-centered EHR use skills in clinical practice.	√	√	√				15
Junod Perron et al. (2014)	Switzerland, Geneva University Hospitals	A 6-month faculty development programme consisted of 4–5 small group sessions (6–7 h in total) and 2 individual coaching sessions (2 h in total)	28 clinical supervisors	Information provider and coach	Mixed methodology, One group, Pretest-posttest, delayed post-test	Increase in participants' communication skills teaching.	√	√	√				11

Schaefer et al. (2014)	USA, Northwestern University Feinberg School of Medicine	An 8-h multimodality training course featuring deliberate practice and feedback	17 clinical faculty members	Information provider and coach	Randomised controlled study, Pretest-posttest	Significant increases in participants' cardiac physical examination interpretation skills.			√				13.5
Tricio et al. (2017)	Chile, University of the Andes, School of Dentistry	A year-long faculty development workshops in student-centered teaching methodologies	176 dental faculty members	Information provider and coach-Facilitator of learning and mentor	One group, Pretest-posttest	Significant decrease in teacher-focused approaches for faculty members from before to after the workshops.	√	√	√				11.5
Junod Perron et al. (2014)	Switzerland, Geneva University Hospitals	A six- to nine-month faculty development program comprising four to five 90-minute small group modules and two 60-minute individual coaching sessions	48 clinical supervisors, 16 from the inpatient setting and 12 from the outpatient setting	Information provider and coach-Facilitator of learning and mentor	Non-equivalent control group, Pretest-posttest	No increase in the number of communication skills recognised by supervisors but significant increase in the number of communication issues discussed interactively in feedback sessions.		√	√				12

Rodrigue et al. (2013)	USA, University of Queensland, School of Medicine	A faculty development curriculum consisted of 5 online modules	38 faculty members	Information provider and coach-Facilitator of learning and mentor	One group, Pretest-posttest	Statistically significant decrease in the number of faculty who reported that the program was not at all effective in providing tools to develop skills and habits.	√	√					10.5
Wong & Fang (2012)	China, Zhejiang University School of Medicine	Two-week faculty development program comprising eight small-group seminars based on the Stanford Faculty Development Program model	28 early to mid-level medical faculty educators	Information provider and coach-Facilitator of learning and mentor-Assessor and diagnostician	One group, Pretest-posttest	Significant improvements in participants' self-reported teaching skills in both global areas of teaching competence as well as in the frequency of employing certain teaching behaviors associated with effective teaching.	√	√	√	√			9.5
Skeff et	USA,	1-month of	12	Information	3	Significant	√	√	√	√			11

al. (1992)	Stanford University Medical Center	facilitator training at the Stanford Faculty Development Program	facilitators and 107 faculty from 12 institutions	provider and coach- Facilitator of learning and mentor- Assessor and diagnostician	cohorts , Pretest-posttest	improvements in participants' self-reported teaching performance.							
Klein et al. (2013)	USA, Cincinnati Children's Hospital Medical Center	A year-long national online faculty development activity on Facebook	17 academic pediatric faculty from 17 different institutions across 12 states and 4 time zones	Scholar and researcher	Mixed metho dology , One group, Pretest-posttest, delayed post-test	Significant increase in participants' self-reported professional Facebook usage.	√	√	√	√			12.5
Pinheiro et al. (2002)	USA, Michigan State University College of Osteopath ic Medicine	A yearlong part-time faculty development fellowship program	54 community-based physician faculty	Information provider and coach- Facilitator of learning and mentor- Assessor and diagnostician - Curriculum developer and implementer - Scholar and researcher- Professional	Mixed metho dology , One group, Pretest-posttest, delayed post-test	Significant increase in participants' self-reported use of educational concepts and greater participation in the educational activities of their home institutions; changes in participants'	√	√	√	√	√		10

						perceptions as medical educators and positive impact on the educational process in their home institutions.							
Shields et al. (2007)	USA, Harvard Medical School	Three two-hour faculty development sessions before the course, and a weekly faculty meeting during the course	70 tutors (Gastroenterology Faculty and attendings or residents in other medical or surgical specialties)	Information provider and coach-Facilitator of learning and mentor	3 cohorts, Pretest-posttest	The tutor as a discussion leader had a significant and positive impact on learning in tutorials, achieving course objectives, improving overall course satisfaction, and increasing a standardized national exam's mean score.	√	√	√	√	√	√	13
Green et al. (2003)	USA, Yale University School of Medicine	The 3-hour faculty development workshop	26 university-based and community-based	Information provider and coach-Facilitator of learning and	One group, Pretest-posttest, delayed post-	Significant improvements in participants' self-reported	√	√	√	√	√		11.5

			general medicine faculty	mentor- Assessor and diagnostician - Role model	test	ambulatory precepting and primary care genetics skill.							
Salerno et al. (2003)	USA, Tripler Army Medical Center	Three 90-minute ambulatory faculty development seminars on written feedback	9 faculty members and 44 third-year students	Information provider and coach- Facilitator of learning and mentor- Assessor and diagnostician	Mixed methodology, One group, Pretest-posttest	Significant improvements in the quantity and quality of written feedback delivered in the ambulatory setting.		√	√	√			11.5
Chung et al. (2012)	Korea, Korean Society for Simulation in Healthcare	A 3-day international, collaborative, multi-professional faculty development program on Simulation-Based Healthcare Education	30 faculty teachers	Information provider and coach- Facilitator of learning and mentor- Assessor and diagnostician - Curriculum developer and implementer- Role model	One group, Pretest-posttest	Significant improvements in participants' self-reported knowledge, skills, and attitudes (KSA) toward simulation teaching.	√	√	√				11
Schlair et al. (2017)	USA, Montefiore Medical Center	The 3-hour longitudinal faculty development program	36 faculty members and 62 residents	Assessor and diagnostician	Mixed methodology, One group,	Significant improvements in participants' feedback quality.	√	√	√				10.5

					Pretest-posttest								
McAndrew et al. (2013)	USA, New York University College of Dentistry	A five-year Excellence in Clinical Teaching Program	59 dental faculty members	Information provider and coach-Facilitator of learning and mentor	8 cohorts, Pretest-posttest, delayed post-test	Significant improvements in participants' self-assessment of their teaching ability.	√	√	√	√	√		9.5
Efstathiou et al. (2018)	USA, Harvard Medical School	A formal mentorship program consisted of assignment of mentor pairs, three formal training sessions over the course of nine months, and regular informal meetings throughout the program	23 junior faculty mentees and 91 junior faculty	Facilitator of learning and mentor	Non-equivalent control group, Pretest-posttest, delayed post-test	Significant increase in participants' satisfaction in five of seven domains related to mentoring.	√	√		√			12.5
Eckstrom et al. (2006)	USA, Oregon Health & Science University	A two-year One-Minute Preceptor faculty development workshop	68 internal medicine continuity clinic preceptors	Information provider and coach-Facilitator of learning and mentor	Nonrandomised controlled study,	Improvements in participants' self-reported teaching behaviors		√	√	√			16

			(44 control and 24 intervention faculty)		Pretest-posttest, delayed post-test	following the intervention.							
Nasmith & Franco (1997)	Canada, McGill University	A half-day workshop on minor surgical procedures	25 family medicine faculty members	Information provider and coach	One group, Pretest-posttest, delayed post-test	Increase in the reported number of minor surgical procedures performed and taught, and in faculty members' perceptions of their competence in performing and teaching these procedures.	√	√	√				7
Fornari et al. (2018)	USA, Hofstra Northwell School of Medicine	A 10-month interprofessional faculty development curriculum consisted of small group work, experiential learning, and critical reflection	169 high potential mentors and 61 facilitator leaders	Facilitator of learning and mentor- Role model-Professional	Mixed methodology, two cohorts, Pretest-posttest	Significant increases in participants' perceptions of humanistic teaching skills; significant increases in participants' self-reported mentoring	√	√	√				11.5

						skills.							
Morzinski et al. (2003)	USA, Medical College of Wisconsin	A comprehensive, 2-year, within-institution faculty development program included monthly seminars and mentor involvement	35 full-time family medicine faculty members	Information provider and coach- Facilitator of learning and mentor- Assessor and diagnostician - Curriculum developer and implementer - Scholar and researcher- Professional- Manager and leader	Longitudinal, multi-method study, six cohorts, Pretest-posttest	Significant, positive changes in the program completers' competence, leadership, presentations, and publications, as well as enhanced retention in academic medicine.	√	√	√	√	√		14
Rosenbaum et al. (2005)	USA, University of Iowa Carver College of Medicine	A 3-year teaching scholars program included monthly sessions, teaching videotapes and faculty development projects	24 faculty members	Information provider and coach- Facilitator of learning and mentor- Assessor and diagnostician - Curriculum developer and implementer - Scholar and researcher- Professional-	One group, Pretest-posttest	Substantial increase in program participants' facilitation of teaching skills workshops and development of teaching improvement systems within their home departments.			√	√	√		11.5

				Manager and leader									
Simpson et al. (2004)	USA, Ambulatory Pediatric Association (APA)/Health Resources and Services Administration (HRSA)	Three 2-day workshops over a 12- to 14-month period	107 pediatric educators from across the United States	Information provider and coach-Facilitator of learning and mentor-Scholar and researcher-Manager and leader	Multi-method study, two cohorts, Pretest-posttest	The faculty development program achieved its objectives, with participants leading workshops, impacting faculty development infrastructure, advancing their own careers, and being strategically positioned in leadership roles with the skills to improve primary care education in the ambulatory setting.	√	√	√	√	√		15
Coogle et al. (2016)	USA, Virginia Common	A 10-month, 160-hour interprofessi	26 participants from	Information provider and coach-	Quasi-experimental	Significant gains in perceived self-	√	√	√	√			13

	wealth University	onal faculty development programme in geriatrics	medicine, nursing, pharmacy, physical therapy, occupational therapy, social work, and the allied health professions	Assessor and diagnostician - Curriculum developer and implementer	study with pre-test post-test design	efficacy and changes in academic teaching, career development, and clinical supervision.							
Davis et al. (2013)	USA, University of Oklahoma College of Pharmacy	One semester Education Scholar Active Learning cohort	11 pharmacy faculty	Information provider and coach- Facilitator of learning and mentor	Mixed methodology, One group, Pretest-posttest	Increase in participants' knowledge of, perceptions of, and implementation of active learning strategies in the didactic classroom.	√	√	√				7.5
Blatt et al. (2018)	USA, The George Washington University School of Medicine	A 2-year GW-Gold Humanistic Mentor Development Program consisted of two summer evening	44 faculty (22 physicians and 22 psychosocial professionals) and 180 first-year	Information provider and coach- Facilitator of learning and mentor- Professional	Mixed methodology, One group, Pretest-posttest	Increase in participants' confidence in their humanistic skills; professional and personal growth and continued	√	√	√	√	√	√	14

		meetings and eight Piggyback FD workshops	medical students			professional identity development in both mentors and students.							
Baral et al. (2010)	Nepal, B. P. Koirala Institute of Health Sciences	A three day long “Training on Problem Based Learning” workshops	25 heterogeneous groups of teachers representing medical, dental, nursing and public health schools	Information provider and coach-Facilitator of learning and mentor	One group, Pretest-posttest	Significant gain in participants’ knowledge of and perceptions of development and dissemination of PBL manual.	√	√	√				9
Reader et al. (2015)	USA, Montefiore Medical Center/Albert Einstein College of Medicine	26 half-day workshops over a 12- to 14-month period	10 busy clinician educators	Scholar and researcher	Mixed methodology, three cohorts, Pretest-posttest, delayed post-test	Increase in participants’ confidence, knowledge and skills to conduct educational research.	√	√	√				11.5
Skeff et al. (1999)	USA, five regions of United States	Five regional 1- to 2-day teaching-improvement workshops	282 university and community faculty	Information provider and coach-Facilitator of learning and	Five cohorts, Pretest-posttest	Increase in participants’ knowledge, skills, and attitudes	√	√	√				10

				mentor		regarding office-based teaching; statistically significant changes in participants' behaviors pertaining to fostering a positive learning climate, communicating goals, and providing feedback, as well as general teaching ability.							
Thampy & Bond (2016)	UK, University of Manchester, Manchester Medical School	Two 90-min contrasting teaching CPD workshops within a full-day tutor conference	73 GP tutors	Information provider and coach-Facilitator of learning and mentor	Non-equivalent control group, Pretest-posttest	Statistically significant rises in teaching ideas post-intervention; tutors who used unstructured discussions generated more ideas	√	√					9

						and reported greater intention to change future teaching practice than those guided by PBL-based vignettes.							
Lemoine et al. (2015)	USA, Louisiana State University Health Sciences Center	An 8-month interprofessional faculty development in simulation-based education consisted of 12-module curriculum	215 health professions education faculty	Information provider and coach- Facilitator of learning and mentor- Assessor and diagnostician - Curriculum developer and implementer	Four cohorts, Pretest-posttest	High faculty receptivity, statistically significant gains for all targeted knowledge, skills, and perspectives, and highly effective overall implementation.	√	√	√				12.5
Williams et al. (2008)	USA, University of Michigan	A four-year faculty development seminar in geriatrics	15 faculty from seven surgical and related disciplines and five medical subspecialties	Information provider and coach- Facilitator of learning and mentor- Assessor and diagnostician - Curriculum developer	Mixed methodology, three cohorts, Pretest-posttest, delayed post-	Changes in attitudes and knowledge of faculty participants; expanded curricula and teaching activities in geriatrics in	√	√	√	√	√		13

				and implementer - Scholar and researcher- Professional-	test	participating departments, and enhanced and altered career trajectories of faculty participants.							
Kuhnigk et al. (2013)	Germany, Medical Faculty of Hamburg University	Four-year teacher training workshops	322 faculty members	Information provider and coach- Facilitator of learning and mentor- Assessor and diagnostician	Four cohorts, Pretest-posttest, delayed post-test	Significant increases in participants' self-assessment of didactic skills up to four years after attending a faculty development workshop.	√	√	√				11
Weppner et al. (2017)	USA, University of Michigan	A year-long interprofessional faculty development curriculum included teaching seminars, journal clubs and teaching observations	35 faculty members included internal medicine and related subspecialties, pharmacy, nurse practitioner, psychology	Information provider and coach- Assessor and diagnostician	One group, Pretest-posttest	Increase in the number of interprofessional copresentations; increased presentation to interprofessional groups and increased cross-profession feedback.			√				8.5

			and psychiatry											
Mkony et al. (2012)	Tanzania, Muhimbili University of Health and Allied Sciences	A 3-day workshop on competency-based education	97 faculty members	Information provider and coach- Facilitator of learning and mentor- Assessor and diagnostician - Curriculum developer and implementer	Two cohorts, Pretest-posttest	Increase in faculty confidence to a level near 'very confident' for items related to explaining, teaching, and assessing competencies; increase in confidence for developing curriculum.		√						9
Hatem et al. (2007)	USA, University of Massachusetts Medical School	A 5-day faculty development workshop on teaching the medical interview	79 medical school faculty	Information provider and coach- Facilitator of learning and mentor-	One group, Pretest-posttest	Significant changes in mean scores of participants' confidence in interview, clinical, teaching and self-awareness skills.	√	√	√					11
Davis et al. (2015)	USA, Emory University	A 2-hour facilitator training session in inter-	53 faculty from the schools of medicine and	Information provider and coach- Facilitator of learning and	Quasi-experimental study with	Significant changes in participants' self-concept for the	√	√	√					13.5

		professional education	nursing	mentor-	pre-test post-test design	knowledge, skills and attitudes toward inter-professional teamwork.							
McCurdy et al. (2004)	USA, The University of Nebraska Medical Center	A year-long course included eight 3-hour workshop sessions	22 faculty from the colleges of Medicine, Dentistry, Nursing and Pharmacy	Manager and leader	Mixed methodology, One group, Pretest-posttest	Statistically significant changes in participants' knowledge about leadership competencies; three themes arose from the analysis of projects: change, management, and interpersonal communications.			√				10
Kesselheim et al. (2019)	USA, The American Society of Hematology (ASH) Medical Educators Institute	A curriculum blending in-person training, which took the form of a 3.5- day intensive workshop,	20 faculty from 20 different institutions	Information provider and coach- Facilitator of learning and mentor- Assessor and diagnostician - Curriculum	One group, Pretest-posttest	Significant increase in participants' self-reported confidence in the knowledge, skills, and attitudes	√	√	√				10

		with follow-up distance learning in the form of a webinar series		developer and implementer - Scholar and researcher- Professional-Manager and leader		targeted by the curriculum.							
Podrazik et al. (2008)	USA, University of Chicago	The faculty development programme consisted of twelve 4-hour sessions	29 hospitalist and general internal medicine faculty members	Information provider and coach- Facilitator of learning and mentor- Assessor and diagnostician	Three cohorts, Pretest-posttest	Significant improvements in participants’ knowledge, attitudes to geriatrics, and perceived behavior in teaching and practicing geriatrics skills.	√	√	√	√			10
Evans et al. (2014)	USA, The Stanford Geriatric Education Center	The 12-hour, eight-module core curriculum was implemented during an intensive weeklong program over a three-year period	34 faculty participants from 11 disciplines, including medicine, came from 19 institutions in 12 states	Information provider and coach	One group, Pretest-posttest	Positive effect on participants’ knowledge, skills, and attitudes related to topics in Health Literacy and Ethnogeriatrics (HLE);	√	√	√	√	√		11.5

						positive effect on participants' scores on the Health Beliefs and Attitudes Survey and dissemination of HLE knowledge by participants at their home institutions.							
Baldwin et al. (2017)	USA, The Academic Pediatric Association	A national, three-year, cohort-based certification program focused on fostering educational scholarship	50 faculty members	Scholar and researcher	Mixed methodology, three cohorts, Pretest-posttest, delayed post-test	Significant increase in participants' self-perceived proficiency in four domains (scholarly approach, professional interactions, educational scholarship, and other educational knowledge/skills); significant increases in	√	√	√	√	√		13

						the numbers of peer-reviewed publications, national presentations, and national leadership/ membership positions, as well as in the number of academic promotions.							
Garner et al. (2018)	India, nursing school in Bengaluru	The 8-h faculty development workshop on simulation	87 nurse faculty	Information provider and coach-Facilitator of learning and mentor-Assessor and diagnostician	One group, Pretest-posttest	Significant improvements in total self-efficacy and subscale scores among nurse educators.		√					12.5
Berbano et al. (2006)	USA, Walter Reed Army Medical Center	Seven 2-hour sessions of faculty development included didactic, role-play, and videotaped performance evaluation.	8 internal medicine faculty	Information provider and coach-Facilitator of learning and mentor-Assessor and diagnostician	One group, Pretest-posttest	Improvement in participants' teaching behaviors, specifically in the quality of questions asked and feedback provided.	√	√	√	√			12.5

Nemec & Welch (2016)	USA, Western New England University College of Pharmacy	A 1.5-hour faculty development seminar	24 pharmacy faculty	Assessor and diagnostician	One group, Pretest-posttest	Significant improvement in the quality of in-house MCQ and faculty confidence in constructing new MCQ.			√				12.5
Rust et al. (2006)	USA, Morehouse School of Medicine	A 1-year longitudinal/ modular workshops	113 community-based preceptors and new or established faculty	Information provider and coach- Facilitator of learning and mentor- Assessor and diagnostician - Curriculum developer and implementer - Scholar and researcher- Manager and leader	10 cohorts , Pretest-posttest	Improvement in participants' self-reported before-after competencies in specific academic skills such as teaching, writing, research, and grant writing.	√	√	√	√	√		14
Walsh et al. (2019)	USA, the Brody School of Medicine, East Carolina University	The 15-Month faculty development program included six 2-day learning	27 clinical faculty members	Information provider and coach- Facilitator of learning and mentor- Curriculum developer	Equivalent control group, Pretest-posttest, delayed post-test	Significant increase in participants' self-assessed knowledge of quality improvement, patient safety,	√	√	√	√			12

		sessions on the principles and application of health systems science		and implementer- - Scholar and researcher- Professional- Manager and leader		adult education principles, and curricular development; significant increase in participants' self-assessed knowledge and confidence in teaching and applying health systems science principles at 1 year post training.							
Phillips et al. (2019)	USA, Chamberlain College of Nursing, Chamberlain University	A 2-hour eLearning faculty development course in clinical teaching included 8 modules	237 clinical nurse educators	Information provider and coach- Facilitator of learning and mentor- Assessor and diagnostician	Mixed methodology, One group, Pretest-posttest, delayed post-test	Significant increase in participants' self-reported knowledge and skill acquisition including transfer of new skills to the nurse educator role at 3 months after	√	√	√	√			10.5

						completing the course.							
Yoon et al. (2016)	Mongolia, The Mongolian National University of Medical Sciences	The 17-day faculty development workshops over 3 weeks	325 faculty members	Information provider and coach-Facilitator of learning and mentor-Assessor and diagnostician - Curriculum developer and implementer- - Scholar and researcher-Manager and leader	Seven groups, Pretest-posttest	Significant improvements in participants' attitudes and perspectives after the educational programs.	√	√					9.5
Dellinges & Curtis (2017)	USA, School of Dentistry, University of California	One-hour training workshop	24 dental school faculty members	Assessor and diagnostician	Randomised controlled study, Pretest-posttest	Significant improvement in MCQ item quality in the intervention group.			√				12.5
Safavi & Sadeghi (2017)	Iran, Hamedan University of Medical Sciences	7 one-week training-consulting courses with a supervised	158 faculty members	Curriculum developer and implementer	Seven cohorts, Pretest-posttest	Significant increase in the number of standard lesson plans developed by	√	√	√				13.5

		and feedback on experience strategy				participants.							
Derakhshan et al. (2015)	Iran, Qazvin University of Medical Sciences	5 educational workshops	faculty members from 7 departments	Assessor and diagnostician	Quasi-experimental study with pre-test post-test design	Significant improvement in MCQ item quality after the educational programs.			√				13.5
