# Supplementary material for: A systematic review of faculty development programs based on the Harden teacher’s role framework model
Source: BMC Med Educ. 2023 Nov 30;23:910. doi: 10.1186/s12909-023-04863-4 (PMC10690997; doi:10.1186/s12909-023-04863-4)
Supplement: Supplementary file 4 — Additional file 4: Appendix 3. The study findings based on the Harden teacher’s role framework. [file 12909_2023_4863_MOESM4_ESM.pdf]

Appendix 3. The study findings based on the Harden teacher's role framework.

The teacher as an information provider and coach

With reference to Harden's domains, the one most covered by the faculty development programs is an information provider and coach, which can be identified in 91 studies [1-91]. Of these, the majority were in the form of workshops (n=32, 35.16%) with various durations [5, 8, 9, 11-13, 20, 25, 28, 33, 34, 36, 39, 41-43, 45, 46, 48, 54, 57-59, 64, 68, 69, 72-75, 85, 87]. Nearly 16.5% of the 91 interventions were described as a short course [1, 2, 6, 18, 19, 22, 23, 50, 55, 56, 70, 78, 80, 81, 91] and fifteen as a seminar series [15, 21, 24, 35, 38, 40, 44, 49, 60, 63, 77, 82, 83, 89, 90]. In two studies [14, 47], the use of a mentorship program was described. There were two fellowship studies [37, 71]. Twenty (21.97%) were described as a longitudinal program [3, 4, 7, 10, 16, 17, 27, 29, 30, 32, 52, 53, 61, 65-67, 76, 84, 86, 88]. Pharmacy, nursing, medical, and dental faculty as well as basic science and clinical science teachers from different departments participated in these studies.

The majority of studies (n=58, 63.73%) used a single-group with a pre- and post- test design [2, 4-6, 13-22, 28, 30-32, 35-37, 42, 44-50, 54-56, 58-60, 62-65, 68, 70-74, 76-79, 81, 82, 84, 86-91]. There were 19 (20.87%) cohort studies with pre-test post-test design [1, 8, 9, 11, 12, 24, 29, 38-41, 43, 51-53, 61, 66, 69, 75]. Eleven studies out of 91 were nonrandomised controlled study with pre-test post-test design [3, 7, 10, 23, 25-27, 33, 34, 57, 67] and there were three randomized controlled studies [80, 83, 85]. One author employed a qualitative methodology only [76], though 11 (12.08%) researchers used a mixed methodology in their work [2, 30, 36, 40, 43, 44, 47, 60, 71, 86, 91].

Although most studies (n=50, 54.94%) used self-report data to assess program outcomes [3, 4, 8, 11-17, 19-21, 23, 24, 28, 30, 32, 33, 37, 41, 42, 45, 50, 52, 53, 55, 58, 59, 61, 63, 65-69, 71, 72, 74-77, 81-83, 87-91], three studies [46, 51, 80] used objective data only. In 38 studies, in addition to self-reported data, objective data sources (e.g., expert opinion, student or resident ratings, student exam scores) were also used [1, 2, 5-7, 9, 10, 18, 22, 25-27, 29, 31, 34-36, 38-40, 43, 44, 47-49, 54, 56, 57, 60, 62, 64, 70, 73, 78, 79, 84-86]. Surveys were the most common method of data collection.

Program outcomes were largely self-reported and were most often measured using surveys. The majority of outcomes reported were at level 2B (83.51%), followed by level 1 (76.92%), level 2A (74.72%) and level 3 (51.64%). Far fewer outcomes are reported for levels 4A (18.68%) and 4B (4.39%). At level 1, seventy studies assessed participants' reactions, which included participants' satisfaction, perceptions of program usefulness, and views on the learning experience, its organization, presentation, content, teaching methods, and quality of instruction [1-6, 8-17, 20, 22-25, 28-33, 35-41, 43-45, 47, 49-55, 58, 59, 61, 64-68, 71, 73-78, 81-87, 89-91]. The majority of participants reported a high level of satisfaction. They also valued the content of programs and particularly appreciated sessions on improving teaching skills. Changes in the attitudes or perceptions among participants (level 2A) was reported by sixty eight articles [1-5, 8-20, 23-25, 27-30, 32, 33, 35, 36, 38-43, 45, 47-50, 52, 54-61, 64-67, 69, 71-76, 78, 81, 82, 84-86, 90, 91]. Most programs led to self-reported changes in participants' attitudes about and perceptions of teaching and learning. In one study [40], faculty reported increased enthusiasm to teach geriatrics, and attitudes toward teaching about and caring for older patients; another study [50] reported significant increases in participants' perceptions of the importance of four online teaching practices. Shealy et al. [19] reported

significant improvement in participants' self-reported self-awareness and self-confidence related to coaching. 76 articles [1-11, 13, 14, 17-19, 21, 23-32, 34, 35, 37-41, 43-49, 51-61, 63, 65-68, 70-74, 76-82, 85-91] evaluated outcomes at level 2B (Modification of knowledge or skills). The majority of faculty development programs led to significant increases in faculty members' cognitive knowledge and skills of different aspects of the teaching-learning process and coaching such as educational concepts and principles, teaching strategies, methods and techniques. Participants also reported an improvement in their teaching and coaching skills such as teaching communication skills, clinical teaching, office-based teaching, giving effective feedback and promoting reflection. Sherbino et al. [2] reported improvements in participants' knowledge about ED-specific (Emergency Department) teaching strategies, and this improvement is maintained at one month. Wong et al. [87] reported significant improvements in participants' small-group teaching skills. In another study [11], participants' ratings of their abilities before and after the seminar revealed statistically significant changes in behaviors pertaining to fostering a positive learning climate, communicating goals, and providing feedback, as well as general teaching ability. Significant changes in faculty participants' OSTE scores and ratings were reported in one study [51]. In another [43], faculty members agreed or strongly agreed that the training strengthened their ability to provide well-rounded, criterion-based justification for grade deductions in professional judgment. They also reported that the faculty development program positively impacted their confidence in discussing professional judgment with students in clinic and enhanced their ability to consistently communicate in a respectful manner with students regarding professional judgment. Forty seven studies measured outcome at level 3 [1-10, 12, 13, 16, 19, 22, 24, 25, 32, 34, 35, 38-40, 43, 44, 47, 52, 55-58, 60-62, 64-67, 71, 72, 77, 78, 82, 83, 88, 90, 91]. Most

programs led to significant improvements in participants' self-reported teaching behaviors, including improved teaching performance, better communication with learners and enhanced feedback quality. Teachers also reported changes in specific teaching methods (e.g., simulation teaching, office-based teaching, small-group teaching, online teaching), use of active learning strategies and fostering a positive learning climate. In one study [65] participants felt that their performance had improved in all 10 teaching behaviors after the intervention. Another study [16] reported significant increases in instructors' self-reported ability to teach pharmacogenomics to pharmacy students. Wong & Fang [90] reported significant improvements in participants' self-reported teaching skills in both global areas of teaching competence as well as in the frequency of employing certain teaching behaviors associated with effective teaching. Moreover, several studies reported observed behavioral changes. In one study [62], students reported changes in shadowing behavior of preceptors; in another [78] peer-, self-, and expert-assessment indicated a transfer of learning into teaching performance. Gardner et al. [22] reported that residents observed improvements in structured teaching behavior among participating faculty. Another study [7] reported significant increases in participants' use of 2 teaching methods (priming and feedback). Changes in the system/organizational practice (level 4A) was reported by seventeen articles [1, 4, 8, 9, 34, 38-40, 43, 47, 52, 58, 61, 71, 72, 78, 88]. For example, Green [72] found that participants reported fully or partially implemented many changes in their clinical practice, podiatric medical teaching, and administrative duties; Rosenbaum et al. [88] reported a substantial increase in program participants' facilitation of teaching skills workshops and development of teaching improvement systems within their home departments. Another study reported the faculty development program achieved its objectives, with participants leading workshops, impacting

faculty development infrastructure, advancing their own careers, and being strategically positioned in leadership roles with the skills to improve primary care education in the ambulatory setting [9]. Change among the participants' students, residents or colleagues (level 4B) was reported only by four studies [1, 34, 43, 47]. In one study [1], the tutor as a discussion leader had a significant and positive impact on learning in tutorials, achieving course objectives, and increasing a standardized national exam's mean score.

The teacher as a facilitator of learning and mentor

Sixty four of faculty development programs focused on the domain of facilitator of learning and mentor [1, 3, 8-12, 14, 15, 17, 22-27, 29, 30, 32, 33, 35, 36, 38-42, 44-50, 52, 54, 55, 57-61, 65, 66, 69-71, 73-76, 78, 81, 84, 85, 87, 88, 90-96]. Of these, the majority were in the form of workshops (n=24, 37.50%) with various durations [8, 9, 11, 12, 25, 33, 36, 39, 41, 42, 45, 46, 48, 54, 57-59, 69, 73-75, 85, 87, 92]. Nearly 14% of the 64 interventions were described as a short course [1, 22, 23, 50, 55, 70, 78, 81, 91] and nine as a seminar series [15, 24, 35, 38, 40, 44, 49, 60, 90]. In three studies [14, 47, 96], the use of a mentorship program was described. There was only one fellowship study [71]. Seventeen (26.56%) were described as a longitudinal program [3, 10, 17, 27, 29, 30, 32, 52, 61, 65, 66, 76, 84, 88, 93-95]. Studies included faculty participants in medicine, nursing, pharmacy and dentistry and as well as basic science and clinical science teachers from different departments.

The majority of articles (n=37, 57.81%) used a single-group with a pre- and post- test design [14, 15, 17, 22, 30, 32, 35, 36, 42, 44-50, 54, 55, 58-60, 65, 70, 71, 73, 74, 76, 78, 81, 84, 87, 88, 90-92, 94, 95]. There were 17 (26.56%) cohort studies with pre-test post-test design [1, 8,

9, 11, 12, 24, 29, 38-41, 52, 61, 66, 69, 75, 93]. Nine studies out of 64 were nonrandomised controlled study with pre-test post-test design [3, 10, 23, 25-27, 33, 57, 96] and there was one randomized controlled study [85]. One author employed a qualitative methodology only [76], though 9 (14.06%) researchers used a mixed methodology in their work [30, 36, 40, 44, 47, 60, 71, 91, 93].

Although most studies (nearly 55% of the 64 interventions) used self-report data to assess program outcomes [3, 8, 11, 12, 14, 15, 17, 23, 24, 30, 32, 33, 41, 42, 45, 50, 52, 55, 58, 59, 61, 65, 66, 69, 71, 74-76, 81, 87, 88, 90, 91, 94, 95], two studies [46, 92] used objective data only. In 27 studies, in addition to self-reported data, objective data sources (e.g., expert opinion, student or resident ratings, student exam scores) were also used [1, 9, 10, 22, 25-27, 29, 35, 36, 38-40, 44, 47-49, 54, 57, 60, 70, 73, 78, 84, 85, 93, 96]. Surveys were the most common method of data collection.

Program outcomes were largely self-reported and were most often measured using surveys. The majority of outcomes reported were at level 2A (87.50%), followed by level 1 and level 2B (82.81% each). Far fewer outcomes are reported for levels 4A (20.31%) and 4B (3.12%). At level 1, fifty three studies assessed participants' reactions [1, 3, 8-12, 14, 15, 17, 22-25, 29, 30, 32, 33, 35, 36, 38-41, 44, 45, 47, 49, 50, 52, 54, 55, 58, 59, 61, 65, 66, 71, 73-76, 78, 81, 84, 85, 87, 90-94, 96]. The majority of participants were satisfied with the programs and rated them as helpful, relevant and useful in providing an opportunity for sharing with other educators. For example, Efstathiou et al. [96] reported a significant increase in participants' satisfaction in five of seven domains related to mentoring. Changes in the attitudes or perceptions among participants (level 2A) was reported by fifty six articles [1, 3, 8-12, 14, 15, 17, 23-25, 27, 29, 30, 32, 33, 35, 36, 38-42, 45, 47-50, 52, 54, 55, 57-61, 65, 66, 69, 71, 73-76,

78, 81, 84, 85, 90, 91, 93-96]. Most programs led to self-reported changes in participants' attitudes about and perceptions of teaching, learning and mentoring. For example, LeBlanc et al. [15] reported a significant increase in the level of comfort in participants' ability to facilitate online case-based discussions; Roos et al. [78] reported that participants felt familiar with learning theory and expressed their intention to apply the learned educational principles; Sullivan et al. [61] reported a major improvement in participants' confidence, commitment to palliative care, and enthusiasm for teaching. 53 articles [1, 3, 8-11, 14, 17, 23-27, 29, 30, 32, 35, 38-41, 44-49, 52, 54, 55, 57-61, 65, 66, 70, 71, 73, 74, 76, 78, 81, 85, 87, 88, 90-95] evaluated outcomes at level 2B (Modification of knowledge or skills). Most participants reported gains in knowledge and skills related to teaching-learning process and mentoring with an emphasis on teaching skills and habits of lifelong learning, providing feedback and the promotion of reflection. Changes in participants' ability to describe the characteristics of a student-centered approach to teaching and the roles and responsibilities of a student mentor were reported in one study [32]. Another study [30] reported an increase in participants' knowledge of, perceptions of, and implementation of active learning strategies in the didactic classroom. Participants also significantly improved in their self-perceived humanistic teaching abilities and mentoring skills over time as assessed by self-report and RN/MD (Registered Nurse/ Medical Doctor) ratings in two OSTE sessions [93]. Thirty one studies measured outcome at level 3 [1, 3, 8-10, 12, 22, 24, 25, 32, 35, 38-40, 44, 47, 52, 55, 57, 58, 60, 61, 65, 66, 71, 78, 88, 90-92, 96]. Most programs led to significant improvements in participants' self-reported use of learner-centered teaching approaches (e.g., development and dissemination of PBL (Problem-Based Learning) manual) and mentoring skills. Teachers also reported improvement in their teaching behaviors, specifically in the quality of questions asked and

feedback provided [55]. Moreover, several studies reported observed behavioral changes. In one study [92], although more of the participants attending mini-CEX (mini-clinical evaluation exercise) workshops provided recommendations as feedback, those without participation spent more time giving feedback and engaged residents' reflection more often; in another [35], the independent teacher evaluations by the housestaff and students indicated improvement in teaching performance of attending physicians. Salerno et al. [44] reported that medical students observed significant improvements in the quality of verbal feedback delivered in the ambulatory setting. Changes in the system/organizational practice (level 4A) was reported by thirteen articles [1, 8, 9, 38-40, 47, 52, 58, 61, 71, 78, 88]. For example, Pinheiro et al. [71] found that participants reported use of educational concepts and greater participation in the educational activities of their home institutions. They also reported changes in participants' perceptions as medical educators and positive impact on the educational process in their home institutions. Green et al. [58] reported significant improvements in participants' self-reported ambulatory precepting and primary care genetics skill. Only two studies [1, 47] assessed change among the participants' students, residents or colleagues (level 4B). One study [47] provided evidence that a development program for interprofessional mentors working within a humanistic framework was successful in developing humanistic interprofessional communities of practice, resulting in both mentors and students experiencing professional and personal growth and continued professional identity development. In another study [1], the students reported that the tutorial made a more important contribution to their learning.

The teacher as a curriculum developer and implementer

Eighteen of faculty development programs focused on the domain of curriculum developer and implementer [10, 29, 34, 38-40, 45, 49, 67, 69, 71, 72, 75, 81, 88, 94, 97, 98]. Of these, the majority were in the form of workshops [34, 39, 45, 69, 72, 75] and longitudinal programs [10, 29, 67, 88, 94, 97] with various durations (n=6, 33.33% each). One of the 18 interventions was described as a short course [81] and three (16.66%) as a seminar series [38, 40, 49]. In one study [98], the use of a mentorship program was described. There was also one fellowship study [71]. Participants in these studies included community-based physician faculty, critical care faculty, academic podiatric physicians and faculty in medicine, nursing, pharmacy, dentistry, psychology, nutrition, audiology, physical therapy and occupational therapy as well as faculty from seven surgical and related disciplines and five medical subspecialties.

The majority of study designs reported were single-group and cohort studies with a pre- and post- test design (38.88% each). Four studies out of 18 were nonrandomised controlled study with pre-test post-test design [10, 34, 67, 98] and there were no randomized controlled studies. None of the reports used a qualitative methodology only, though 2 (11.11%) researchers used a mixed methodology in their work [40, 71].

Although most studies (n=9, 50%) used self-report data to assess program outcomes [45, 67, 69, 71, 72, 75, 81, 88, 94], one study [97] used objective data only. In 8 studies, in addition to self-reported data, objective data sources (e.g., expert opinion, student or resident ratings, student exam scores, multiple-choice questions (MCQs), CV (Curriculum Vitae) review) were also used [10, 29, 34, 38-40, 49, 98]. Surveys were the most popular method of data collection.

Program outcomes were largely self-reported and were most often measured using surveys. The majority of outcomes reported were at level 2A and level 2B (83.33% each), followed by

level 1 (77.77%) and level 3 (55.55%). Fewer outcomes are reported for level 4A (38.88%). Only one study reported outcomes for level 4B. At level 1, fourteen studies assessed participants' reactions [10, 29, 38-40, 45, 49, 67, 71, 75, 81, 94, 97, 98]. As with the other domains, ratings of satisfaction and perceived usefulness of the programs were high. Most participants reported that they would recommend the programs highly to others. Changes in the attitudes or perceptions among participants (level 2A) was reported by fifteen articles [10, 29, 38-40, 45, 49, 67, 69, 71, 72, 75, 81, 94, 97]. Most programs led to significant improvements in participants' attitudes and perspectives after the educational programs. For example, Mkony et al. [69] reported an increase in faculty confidence for developing curriculum; Gates et al. [49] reported significant changes in participants' confidence for a wide range of academic skills such as curriculum design. In the studies that assessed knowledge and skills (level 2B), positive results were shown in both [10, 29, 34, 38-40, 45, 49, 67, 71, 72, 81, 88, 94, 97]. One study [10] reported a significant increase in participants' self-assessed knowledge of quality improvement, patient safety, adult education principles, and curricular development. Another study [97] reported a significant increase in the number of standard lesson plans developed by participants Ten studies measured outcome at level 3 [10, 34, 38-40, 67, 71, 72, 88, 98]. The majority of program outcomes were positive. Windish et al. [98] reported improvement in self-assessed curricular development, implementation, and evaluation skills and enjoyment of participants. Authors from one study [34] reported that as a result of engagement in faculty development programs, 10 faculty members developed 50 instructional Units. Participants in another program incorporated geriatrics teaching into clinical teaching [40]. Changes in the system/organizational practice (level 4A) was reported by seven articles [34, 38-40, 71, 72, 88]. For example, a four-year faculty development

program to enhance geriatrics learning among house officers in surgical and related disciplines and medical subspecialties resulted in expanded curricula and teaching activities in geriatrics in participating departments, and enhanced and altered career trajectories of faculty participants. Moreover, new rotations were established that included a new month-long rotation in a geriatrics rehabilitation setting and 4 half-days in geriatrics outpatient settings [40]. Only one study [34] assessed change among the participants' students, residents or colleagues (level 4B). In this study, students in 9 of the 10 project classes demonstrated significant gains in knowledge of discipline-based concepts and issues in aging.

The teacher as an assessor and diagnostician:

Forty-five of faculty development programs focused on the domain of assessor and diagnostician [3, 5, 8, 12, 17, 22, 24, 25, 29, 32, 35, 38-46, 49, 55, 58, 60, 63, 65-67, 69, 71, 73, 75, 78, 81, 88, 90-92, 99-105]. Of these, the majority were in the form of workshops (n=18, 40%) with various durations [5, 8, 12, 25, 39, 41-43, 45, 46, 58, 69, 73, 75, 92, 99, 103, 104]. Six (13.33%) of the 45 interventions were described as a short course [22, 55, 78, 81, 91, 101] and ten as a seminar series [24, 35, 38, 40, 44, 49, 60, 63, 90, 102]. There was one fellowship study [71]. Nine (20%) were described as a longitudinal program [3, 17, 29, 32, 65-67, 88, 105]. Faculty from the schools of medicine, nursing, pharmacy and dentistry as well as faculty members from the departments of basic, clinical and allied sciences participated in these studies.

The majority of studies (n=26, 57.77%) used a single-group with a pre- and post- test design [5, 17, 22, 32, 35, 42, 44-46, 49, 55, 58, 60, 63, 65, 71, 73, 78, 81, 88, 90-92, 101, 102, 105].

There were 12 (26.66%) cohort studies with pre-test post-test design [8, 12, 24, 29, 38-41, 43, 66, 69, 75]. Six studies out of 45 were nonrandomised controlled study with pre-test post-test design [3, 25, 67, 99, 100, 104] and there was only one randomized controlled study [103]. None of the reports used a qualitative methodology only, though seven researchers used a mixed methodology in their work [40, 43, 44, 60, 71, 91, 105].

Twenty-three studies (nearly 51% of reviewed articles) used self-report data to assess program outcomes [3, 8, 12, 17, 24, 32, 41, 42, 45, 55, 58, 63, 65-67, 69, 71, 75, 81, 88, 90, 91, 100]. Five studies [46, 92, 99, 103, 104] used objective data only. In 17 studies, in addition to self-reported data, objective data sources (e.g., expert-assessment, student or resident ratings, student exam scores, multiple-choice questions, CV review) were also used [5, 22, 25, 29, 35, 38-40, 43, 44, 49, 60, 73, 78, 101, 102, 105]. Surveys were the most common method of data collection.

Program outcomes were largely self-reported and were most often measured using surveys. The majority of outcomes reported were at level 2B (88.88%), followed by level 1 and level 2A (77.77% each) and level 3 (62.22%). Far fewer outcomes are reported for levels 4A (20%). Only one study reported outcomes for level 4B. At level 1, thirty five studies assessed participants' reactions. Overall reactions by faculty participants to the programs were extremely positive. Programs were rated by participants as extremely useful and relevant to clinical and educational activities. Changes in the attitudes or perceptions among participants (level 2A) was reported by thirty five articles [3, 5, 8, 12, 17, 24, 25, 29, 32, 35, 38-43, 45, 49, 55, 58, 60, 65-67, 69, 71, 73, 75, 78, 81, 90, 91, 100, 101, 105]. Most programs led to self-reported changes in participants' self-efficacy and confidence in student assessment. For example, Griffeth & Wiederman [101] reported statistically significant differences in both the

self-rated confidence of faculty members in their ability to accurately rate student performance and in the actual clinical assessment ratings of students after the intervention. One study [65] reported significant increases in hospitalist confidence in giving feedback, receiving feedback, and teaching efficacy. Another study [69] reported increase in faculty confidence to a level near 'very confident' for items related to explaining, teaching, and assessing competencies. 40 articles [3, 5, 8, 17, 24, 25, 29, 32, 35, 38-41, 43-46, 49, 55, 58, 60, 63, 65-67, 71, 73, 78, 81, 88, 90-92, 99-105] evaluated outcomes at level 2B (Modification of knowledge or skills). The majority of faculty development programs led to significant increases in faculty members' cognitive knowledge and skills of different aspects of assessment of students' learning such as development of MCQs, giving effective feedback and workplace-based assessment. In four studies [99, 102-104], effective changes in the learning and performances of medical educators in the development of MCQs were noted. One study [102] reported an improvement to a faculty member's confidence in identifying quality and poor MCQ. Another study [105] reported significant improvements in participants' feedback quality. Participants also reported an increase in their ability to describe the characteristics of the components of assessment in pharmacy education [32]. Twenty eight studies measured outcome at level 3 [3, 5, 8, 12, 22, 24, 25, 32, 35, 38-40, 43, 44, 55, 58, 60, 65-67, 71, 78, 88, 90-92, 100, 101]. Most programs indicated self-reported behavior changes, with a particular focus on improved feedback process and MCQ construction. Moreover, several studies reported observed behavioral changes. In two studies [44, 60], medical students observed significant improvements in the quantity and quality of verbal and written feedback delivered in the ambulatory setting; in another [92] enhancement of giving feedback to residents was observed. Another study [43] revealed the clinical faculty's evaluation of professional judgment during patient care was enhanced by

training. Changes in the system/organizational practice attributable to the educational program (level 4A) were infrequently noted [8, 38-40, 43, 58, 71, 78, 88]. In one study [71], participants reported active involvement in the educational processes of their home institutions. In another study [43], faculty training program enhanced evaluation of students' ethics and professionalism in the academic clinical setting. Participants also reported that they teach the concepts and skills they learned during their programs (e.g., evaluation methods, learner assessment, writing effective test items, giving feedback) to their colleagues [40, 71, 88]. Only one study reported outcomes for level 4B [43]. In this study, students were more positive in outcomes assessments about their competency and learning experiences related to professionalism and ethics.

The teacher as a role model

Five of faculty development programs focused on the domain of role model [58, 78, 81, 93, 106]. Of these, the majority were described as a short course with various durations [78, 81, 106]. One of the 5 interventions was in the form of workshops [58]. There was also one longitudinal program [93]. University-based and community-based general medicine faculty and as well as a mix of medical educators from multiple schools participated in these studies.

The majority of studies (n=3, 60%) used a single-group with a pre- and post- test design [58, 78, 81]. There was only one cohort study with pre-test post-test design [93]. One study out of 5 was nonrandomised controlled study with pre-test post-test design [106]. Two of the researchers used a mixed methodology in their work [93, 106].

Although all studies used self-report data to assess program outcomes [58, 78, 81, 93, 106], in 3 studies, in addition to self-reported data, objective data sources (e.g., expert assessment, OSTEs, multiple-choice questions) were also used [78, 93, 106]. Questionnaires were the most popular method of data collection.

Program outcomes were largely self-reported and were most often measured using questionnaires. The majority of outcomes reported were at level 2A (100%), followed by level 1 and level 2B (80% each). No studies reported outcomes for level 4B. All but one report presented data on the participants' reactions [58, 78, 81, 93]. The majority of participants agreed or strongly agreed that the program was excellent and that they would highly recommend the program to their colleagues. All studies evaluated outcomes at level 2A. Changes in attitudes and perceptions attributable to the educational program were infrequently noted. In one study [93], high potential mentors in both cohorts perceived significant improvement in role model skill during the second assessment compared with the first. Similarly, the standardized RNs/MDs in both cohorts felt that the mentors significantly improved in role model skill. Two of the five studies collected data relating to level 3 outcomes [58, 78]. The majority of program outcomes were positive. Authors from one study [58] reported that as a result of combining clinical content and clinical teaching in faculty development workshops, a majority of participants fully or partially implemented changes in their behavior areas within 3 months. In another study [78], participants tried to be an enthusiastic role model for students and to motivate students to prepare for a complex profession. Two reports of change at the level of impact on the organizational practices were found [58, 78]. Roos et al. [78], reported that their teaching education program supported self-reflection of medical educators in their professional environment, promoted collegiality and

collaboration within and across traditional discipline boundaries, and exerted an important impact on an effective faculty development. No studies assessed change among the participants' students, residents or colleagues (level 4B).

The teacher as a manager and leader

Nineteen of faculty development programs focused on the domain of manager and leader [3, 8-10, 25, 35, 37-39, 45, 49, 53, 75, 78, 88, 94, 107-109]. Of these, the majority were in the form of workshops (n=7, 36.84%) with various durations [8, 9, 25, 39, 45, 75, 107]. Two (10.52%) of the 19 interventions were described as a short course [78, 108] and three as a seminar series [35, 38, 49]. There was only one fellowship study [37]. Six (31.57%) were described as a longitudinal program [3, 10, 53, 88, 94, 109]. Faculty from the colleges of medicine, dentistry, nursing and pharmacy and as well as basic science teachers from different departments and clinical teachers in family medicine, general pediatrics, and general internal medicine participated in these studies.

The majority of studies (n=9, 47.36%) used a single-group with a pre- and post- test design [35, 37, 45, 49, 78, 88, 94, 107, 109]. There were 7 (36.84%) cohort studies with pre-test post-test design [8, 9, 38, 39, 53, 75, 108]. Three studies out of 19 were nonrandomised controlled study with pre-test post-test design [3, 10, 25]. None of the reports used a qualitative methodology only, though authors from one study used a mixed methodology in their work [107].

Ten studies used self-report data to assess program outcomes [3, 8, 37, 45, 53, 75, 88, 94, 107, 108]. In 9 studies, in addition to self-reported data, objective data sources (e.g., student or

resident ratings, student exam scores, multiple-choice questions, peer- and expert-assessment, CV review) were also used [9, 10, 25, 35, 38, 39, 49, 78, 109]. Surveys were the most common method of data collection.

Program outcomes were largely self-reported and were most often measured using surveys. The majority of outcomes reported were at level 2B (94.73%), followed by level 1 (89.47%), level 2A (78.94%) and level 3 (63.15%). Fewer outcomes are reported for level 4A (36.84%). No studies reported outcomes for level 4B. At level 1, seventeen studies assessed participants' reactions to the programs [3, 8-10, 25, 35, 37-39, 45, 49, 53, 75, 78, 94, 108, 109]. In all cases, the overall program was well-rated by participants. They also believed that the content of programs was appropriate and addressed their learning needs. Impact on attitudes or perceptions (level 2A) was reported by fifteen articles [3, 8-10, 25, 35, 38, 39, 45, 49, 75, 78, 94, 108, 109]. Self-reported changes in attitudes and perceptions included enhanced self-efficacy and confidence in collaborative practice, planning and conducting faculty development programs and leadership and management of work teams. Faculty also reported improved confidence in proficiency of clinical quality improvement (QI) concepts. 18 articles evaluated outcomes at level 2B [3, 8-10, 25, 35, 37-39, 45, 49, 53, 78, 88, 94, 107-109]. The majority of faculty development programs led to significant increases in faculty members' cognitive knowledge and skills of different aspects of the educational leadership and management such as time management, change management, small-group leadership, leadership and management of work teams, educational leadership and hospital administration and management. Participants also reported an improvement in their clinical leadership skills and financial skills. In two studies [108, 109], the programs were successful in improving QI knowledge and skill in key QI components. Another study [107], reported statistically

significant changes in participants' knowledge about leadership competencies. Gjerde et al. [37] reported significant improvement in participants' self-reported leadership/advocacy skills. Level 3 results reporting changed behavior were presented in twelve articles [3, 8-10, 25, 35, 38, 39, 78, 88, 108, 109]. Most programs led to significant improvements in participants' self-reported leadership and management behaviors, including improved management performance, applying team principles and health systems science principles into daily work, applying QI into teaching and better collaboration with other professionals. In one study [109] significant improvement was noted in the application of the QI concepts learned during the program. Participants also were successful in proposing and implementing a QI project. In another study [8], participants planed and conducted faculty development workshops for practitioners on basic topics related to office-based precepting (e.g., principles of adult learning, effective and efficient teaching, evaluation, feedback). Changes in the system/organizational practice (level 4A) was reported by seven articles [8, 9, 38, 39, 78, 88, 108]. In one study [8], participants reported success in recruiting more community sites for learners, enhanced relations with community preceptors, and increased formal acknowledgment of the contributions of community preceptors (through awards, access to university resources, etc) at their institutions. One participant in this study now leads the Resident Education and Training Special Interest Group of the American Academy of Pediatrics' Section on Community Pediatrics, which is developing a ''Starter Kit'' to assist practitioners interested in precepting trainees in their offices. Another study reported the faculty development program achieved its objectives, with participants leading workshops, impacting faculty development infrastructure, advancing their own careers, and being strategically positioned in leadership roles with the skills to improve primary care education in

the ambulatory setting [9]. Participants also reported working on another QI project utilizing the tools taught in the course in one study [108]. Rosenbaum et al. [88] reported a substantial increase in program participants' facilitation of teaching skills workshops and development of teaching improvement systems within their home departments. No studies reported outcomes for level 4B.

The teacher as a scholar and researcher

Twenty nine of faculty development programs focused on the domain of scholar and researcher [6, 9, 10, 12, 14, 32, 34, 37-40, 45, 49, 68, 71, 72, 75, 88, 94, 100, 110-118]. Most of these articles were in the form of workshops (n=10, 34.48%) with various durations [9, 12, 34, 39, 45, 68, 72, 75, 115, 117]. Nearly 10.5% of the 29 interventions were described as a short course [6, 113, 116] and three as a seminar series [38, 40, 49]. In three studies [14, 111, 114], the use of a mentorship program was described. There were three fellowship studies [37, 71, 118]. Six (20.68%) were described as a longitudinal program [10, 32, 88, 94, 110, 112]. Participants in these studies included faculty from the schools of medicine, dentistry, nursing and pharmacy and as well as entry level medical teachers and junior/mid-career clinical teachers in family medicine, general pediatrics, critical care and general internal medicine.

The majority of studies (n=16, 55.17%) used a single-group with a pre- and post- test design [6, 14, 32, 37, 45, 49, 68, 71, 72, 88, 94, 112-114, 117, 118]. There were 9 (31.03%) cohort studies with pre-test post-test design [9, 12, 38-40, 75, 110, 111, 115]. Four studies out of 29 were nonrandomised controlled study with pre-test post-test design [10, 34, 100, 116]. None

of the reports used a qualitative methodology only, though authors from six studies used a mixed methodology in their work [40, 71, 110, 112, 115, 118].

Eighteen studies used self-report data to assess program outcomes [12, 14, 32, 37, 45, 68, 71, 72, 75, 88, 94, 100, 110, 111, 113, 116-118]. In 11 studies, in addition to self-reported data, objective data sources (e.g., the number of grant submissions, retention rates, or success in promotion/tenure, the total number of peer reviewed publications by faculty members, CV review) were also used [6, 9, 10, 34, 38-40, 49, 112, 114, 115]. Surveys were the most common method of data collection.

Program outcomes were largely self-reported and were most often measured using surveys. The majority of outcomes reported were at level 2B (89.65%), followed by level 1 (79.31%), level 2A (72.41%) and level 3 (58.62%). Far fewer outcomes are reported for levels 4A (31.03%) and 4B (3.44%). At level 1, twenty three studies assessed participants' reactions to the programs [6, 9, 10, 12, 14, 32, 37-40, 45, 49, 68, 71, 75, 94, 100, 110, 112, 114-117]. The majority of participants were satisfied or highly satisfied with the programs. They also gave high scores to all components of the faculty development programs. Changes in attitudes and perceptions attributable to the educational program was reported by twenty one studies [9, 10, 12, 14, 32, 38-40, 45, 49, 71, 72, 75, 94, 100, 110-112, 115-117]. Self-reported changes in attitudes included greater enthusiasm and motivation as a scholar and researcher, increased confidence to conduct educational research, and enhanced awareness of the value of a scholarly approach to teaching. Participants also reported increased understanding of, and intent to try, evidence-based medicine practice. In one study [45], participants reported that they felt confident in designing and analyzing education research projects following the program. 26 articles measured outcomes at level 2B [6, 9, 10, 14, 32, 34, 37-40, 45, 49, 68, 71, 72, 88, 94,

100, 110-113, 115-118]. The majority of faculty development programs led to significant improvement in participants' self-reported knowledge and skills of different domains of the educational research and scholarship of teaching and learning, such as research design, critical appraisal of the literature, scientific writing, writing grant applications and evidence-based medicine (EBM). Participants also reported an improvement in their technology/informatics skills (e.g., search MEDLINE, use filters while searching, read journals on-line). In three studies [37, 68, 72], participants reported gains in knowledge and skills regarding EBM practice. Improvement in participants' self-reported before-after competencies in specific academic skills such as writing, research, and grant writing was noted in one study [39]. In another study [94], an interactive, seminar-style course using a diverse set of local experts as instructors improved participants' research skills (e.g., modern clinical trial design and implementation, critical appraisal of medical literature, statistical evaluation and modeling of biomedical data, development and submission of a successful grant proposal). Srivastava et al. [118] reported knowledge gained during fellowship program helped participants in guiding educational research at their workplaces. Reader et al. [115] reported an increase in participants' confidence, knowledge and skills in educational scholarship and dissemination. Level 3 results reporting changed behavior were presented in seventeen articles [6, 9, 10, 12, 32, 34, 38-40, 71, 72, 88, 100, 110, 112, 114, 117]. Self-reported behavior changes included increased educational research activities and grant applications, improved critical appraisal of medical literature, use of evidence-based medicine in practice, use of scholarly approach in teaching-learning process and greater involvement in educational scholarship programs. One study [34] reported an increase in research and scholarly activity in the area of aging by faculty participants. Another study [72], reported an improvement in participants' self-reported EBM

practice and EBM teaching skills and, within 3 months, fully or partially implemented many changes in their clinical practice. Manwell et al. [6] reported that an interdisciplinary faculty development program led to an increase in faculty research activities in the alcohol area. In their study, 36% of the participants reported conducting research. There was also an improvement in self-reported competencies in medical education literature critical appraisal and behaviors related to the use of evidence in educational practice [100]. Moreover, several studies reported observed behavioral changes. In one study [6], 10% of the participants had submitted manuscripts for publication and 12% had submitted grant applications. In another [114], significant increases in the total number of peer-reviewed publications for junior faculty protégés were observed, but there were non-significant improvements in the protégés' number of grant submissions, retention rates, or success in promotion/tenure. Nine reports of change at the level of impact on the organization were found [9, 34, 38-40, 71, 72, 88, 110]. Three studies [38, 39, 110], reported significant, positive changes in program completers' competence, national leadership/ membership positions, national presentations, and peer-reviewed publications, as well as enhanced retention in academic medicine. In one study [38], two years after faculty development program completion, 80% of program completers were retained in full-time academic positions, either locally or nationally. In another study [88], postprogram, education-related scholarship also increased as compared to preprogram activities. Specifically, there was a substantial increase in the number of education-related publications and education-related presentations at regional and national meetings. In addition, scholars had increased participation in national educational organizations including attending meetings and membership on committees. Other institutional changes included establishing an educational research group, cross-institutional collaboration and increased emphasis on

educational scholarship in promotion/tenure at medical school [9]. Only one study [34] assessed change among the participants' students, residents or colleagues (level 4B). In this study, faculty members and their students developed a research agenda to investigate topics in aging.

The teacher as a professional

Twenty four of faculty development programs focused on the domain of professional [3, 10, 12, 14, 17, 19, 38, 40, 45, 47, 49, 61, 64, 70, 71, 78, 88, 93-95, 106, 111, 117, 119]. Most of these articles (n=9, 37.5%) were in the form of longitudinal program [3, 10, 17, 61, 88, 93-95, 119]. Four (16.66%) were described as workshops with various durations [12, 45, 64, 117]. Nearly 16.5% of the 24 interventions were described as a short course [19, 70, 78, 106] and three as a seminar series [38, 40, 49]. In three studies [14, 47, 111], the use of a mentorship program was described. There was only one fellowship study [71]. A mix of professions, including medicine, nursing, pharmacy, dentistry, physical therapy, occupational therapy and speech-language pathology as well as junior/mid-career clinical faculty members and community-based physician faculty participated in these studies.

The majority of studies (n=15, 62.5%) used a single-group with a pre- and post- test design [14, 17, 19, 45, 47, 49, 64, 70, 71, 78, 88, 94, 95, 117, 119]. There were 6 (one fourth of the studies reviewed) cohort studies with pre-test post-test design [12, 38, 40, 61, 93, 111]. Three studies out of 24 were nonrandomised controlled study with pre-test post-test design [3, 10, 106]. One author employed a qualitative methodology only [64], though 5 (20.83%) researchers used a mixed methodology in their work [40, 47, 71, 93, 106].

More than half of the studies (54.16%) used self-report data to assess program outcomes [3, 12, 14, 17, 19, 45, 61, 71, 88, 94, 95, 111, 117]. In 11 studies, in addition to self-reported data, objective data sources (e.g., retention rates, or success in promotion/tenure, engagement index, CV review) were also used [10, 38, 40, 47, 49, 64, 70, 78, 93, 106, 119]. Surveys were the most common method of data collection.

Program outcomes were largely self-reported and were most often measured using surveys. The majority of outcomes reported were at level 2A (87.5%), followed by level 2B (83.33%), level 1 (75%) and level 3 (58.33%). Fewer outcomes were reported for levels 4A (29.16%) and 4B (4.16%). Where reaction was assessed [3, 10, 12, 14, 17, 38, 40, 45, 47, 49, 61, 64, 71, 78, 93, 94, 117, 119], many program components were found to be of value: the interaction with colleagues and peers; the instructional design used and the creation of positive learning environments. Changes in attitudes and perceptions attributable to the educational program was reported by twenty one studies [3, 10, 12, 14, 17, 19, 38, 40, 45, 47, 49, 61, 64, 71, 78, 93-95, 106, 111, 117]. Self-reported changes in attitudes included greater enthusiasm and motivation for lifelong learning, development or strengthening of responsibility for teaching, and an increased sense of community and collegiality. Participants also reported an increased understanding of individual professional development, career development, professional responsibility and interpersonal relationships. 20 articles measured outcomes at level 2B [3, 10, 14, 17, 19, 38, 40, 45, 47, 49, 61, 70, 71, 78, 88, 93-95, 111, 117]. The majority of faculty development programs led to significant improvement in participants' self-reported knowledge, skills, and attitudes in the domains of professional development and scholarship, including the ability to write career goals, align activities with those goals and in the number of and amount of time spent pursuing activities related to those goals. Participants also reported

an improvement in their professional skills (e.g., setting career goals and career planning, obtaining work-life balance, assessing professional development needs, resolving conflict and negotiation, writing curriculum vitae and portfolio development). Baker et al. [95] reported significant improvements in the areas of knowledge, skills, community, and feelings of success and confidence about the principles of IPE (Interprofessional Education) and faculty development. Improvements in meeting faculty needs related to professional development, individual feedback and career development were noted in one study [14]. In another study [94], a novel broad-based career development course improved professional skills of critical care medicine trainees (e.g., balancing personal and professional life, identification and prevention of burnout, developing a curriculum vitae). Gates et al. [49] reported an increase in participants' knowledge and skills in legal issues in the educational environment. Fourteen of the twenty-four studies gathered data on level 3 outcomes [3, 10, 12, 19, 38, 40, 47, 61, 64, 71, 78, 88, 117, 119]. Most participants expressed intention to change their behavior as a result of participating in the programs. Researchers from one study [119] reported that as a result of engagement in a faculty development program, qualitative and quantitative metrics for engagement and professional growth improved dramatically during the implementation period. In this study, faculty development program resulted in more highly engaged and productive faculty members who were more likely to remain long term within the college. Another study [19], reported an improvement in participants' ability to manage professional relationships. Additionally, faculty members described beneficial effects in their individual professional development, development of a shared mental model of professional responsibility, and interpersonal relationships [64]. In one study [12], participants reported that the greatest changes in their current teaching practices occurred in their ability to design, implement, and

evaluate a course of instruction, thus increasing their perceptions of progress as a faculty member. In another [3], program participants had significantly greater pre–post-change scores than nonparticipants for professional outcomes (e.g., self-directed learning, working in groups, time management, and administration). Seven studies reported changes at the institutional level [38, 40, 47, 61, 71, 78, 88]. In one study [61], program participants reported increases in proportion of time spent in palliative care practice. Participants also reported having accomplished palliative care-related professional development activities since course completion (e.g., additional training, application for certification, grant submission). In another study [78], teaching performance ratings improved in students' evaluations. Changes in participants' perceptions as medical educators and positive impact on the educational process in their home institutions were noted in one study [71]. Other institutional changes included enhanced retention of program completers in academic medicine and increased professional participation and use of professional academic skills among participants in the educational activities of their home institutions. Only one study [47] assessed change among the participants' students, residents or colleagues (level 4B). This study led to the development of humanistic interprofessional communities of practice, resulted in both mentors and students experienced professional and personal growth and continued professional identity development.

References

1. Shields HM, Guss D, Somers SC, Kerfoot BP, Mandell BS, Travassos WJ, Ullman SM, Maroo S, Honan JP, Raymond LW *et al*: **A faculty development program to train tutors to be discussion leaders rather than facilitators**. *Acad Med* 2007, **82**(5):486-492.

2. Sherbino J, Frank J, Lee C, Bandiera G: **Evaluating "ED STAT!": A Novel and Effective Faculty Development Program to Improve Emergency Department Teaching**. *Academic Emergency Medicine* 2006, **13**(10):1062-1069.
3. Cole KA, Barker LR, Kolodner K, Williamson P, Wright SM, Kern DE: **Faculty Development in Teaching Skills: An Intensive Longitudinal Model**. *Academic Medicine* 2004, **79**(5):469-480.
4. Evans KH, Bereknyei S, Hikoyeda N, Tzuang M, Braddock CH: **The impact of a faculty development program in health literacy and ethnogeriatrics**. *Academic Medicine* 2014, **89**(12):1640-1644.
5. Quirk ME, DeWitt T, Lasser D, Huppert M, Hunniwell E: **Evaluation of primary care futures: A faculty development program for community health center preceptors**. *Academic medicine* 1998, **73**(6):705-707.
6. Manwell LB, Pfeifer J, Stauffacher EA: **An interdisciplinary faculty development model for the prevention and treatment of alcohol use disorders**. *Alcoholism: Clinical and Experimental Research* 2006, **30**(8):1393-1399.
7. Lye P, Heidenreich C, Wang-Cheng R, Bragg D, Simpson D: **Experienced clinical educators improve their clinical teaching effectiveness**. *Ambulatory Pediatrics* 2003, **3**(2):93-97.
8. Roberts KB, Devries JM: **APA/HRSA National Faculty Development Scholars Program: Community-Based Teaching Track**. *Ambulatory Pediatrics* 2004, **4**(1 SUPPL.):92-97.
9. Simpson DE, Bragg D, Biernat K, Treat R: **Outcomes results from the evaluation of the APA/HRSA Faculty Scholars Program**. *Ambulatory Pediatrics* 2004, **4**(1 SUPPL.):103-112.
10. Walsh DS, Lazorick S, Lawson L, Lake D, Garrison HG, Higginson J, Vos P, Baxley E: **The Teachers of Quality Academy: Evaluation of the Effectiveness and Impact of a Health Systems Science Training Program**. *American Journal of Medical Quality* 2019, **34**(1):36-44.
11. Skeff KM, Stratos GA, Bergen MR, Sampson K, Deutsch SL: **Regional teaching improvement programs for community-based teachers**. *American Journal of Medicine* 1999, **106**(1):76-80.
12. Mitcham MD, Lancaster CJ, Stone BM: **Evaluating the effectiveness of occupational therapy faculty development workshops**. *American Journal of Occupational Therapy* 2002, **56**(3):335-339.
13. Assemi M, Mutha S, Hudmon KS: **Evaluation of a train-the-trainer program for cultural competence**. *American Journal of Pharmaceutical Education* 2007, **71**(6).
14. Haines SL, Popovich NG: **Engaging external senior faculty members as faculty mentors**. *American Journal of Pharmaceutical Education* 2014, **78**(5):1-6.
15. LeBlanc JM, Pruchnicki MC, Rohdieck SV, Khurma A, Dasta JF: **An instructional seminar for online case-based discussions**. *American Journal of Pharmaceutical Education* 2007, **71**(3).
16. Lee KC, Ma JD, Hudmon KS, Kuo GM: **A train-the-trainer approach to a shared pharmacogenomics curriculum for US colleges and schools of pharmacy**. *American Journal of Pharmaceutical Education* 2012, **76**(10):1-8.
17. Popovich NG, Peverly SL, Jackson TR: **Effectiveness of a weekly faculty conversation forum about teaching**. *American Journal of Pharmaceutical Education* 2006, **70**(5):1-7.

18. S.M S, B.S F, S.E D, L.G M: **A 1-day course to improve the teaching effectiveness of health professions faculty members**. *American journal of pharmaceutical education* 2012, **76**(1):1-9.
19. Shealy SC, Worrall CL, Baker JL, Grant AD, Fabel PH, Walker CM, Ziegler B, Maxwell WD: **Assessment of a faculty and preceptor development intervention to foster self-awareness and self-confidence**. *American Journal of Pharmaceutical Education* 2019, **83**(7):1534-1546.
20. Paige JT, Arora S, Fernandez G, Seymour N: **Debriefing 101: Training faculty to promote learning in simulation-based training**. *American Journal of Surgery* 2015, **209**(1):126-131.
21. Wong JG, Son D, Miura W: **Cross-Cultural Interprofessional Faculty Development in Japan: Results of an Integrated Workshop for Clinical Teachers**. *American Journal of the Medical Sciences* 2017, **354**(6):597-602.
22. Gardner AK, Timberlake MD, Dunkin BJ: **Faculty Development for the Operating Room: An Examination of the Effectiveness of an Intraoperative Teaching Course for Surgeons**. *Annals of Surgery* 2019, **269**(1):184-190.
23. Davis BP, Clevenger CK, Posnock S, Robertson BD, Ander DS: **Teaching the teachers: Faculty development in inter-professional education**. *Applied Nursing Research* 2015, **28**(1):31-35.
24. Skeff KM, Stratos GA, Berman J, Bergen MR: **Improving clinical teaching. Evaluation of a national dissemination program**. *Archives of internal medicine* 1992, **152**(6):1156-1161.
25. Ebrahimi S, Kojuri J: **Assessing the impact of faculty development fellowship in Shiraz University of medical sciences**. *Archives of Iranian Medicine* 2012, **15**(2):79-81.
26. Lye PS, Simpson DE, Wendelberger KJ, Bragg DS: **Clinical teaching rounds. A case-oriented faculty development program**. *Archives of pediatrics & adolescent medicine* 1998, **152**(3):293-295.
27. Junod Perron N, Nendaz M, Louis-Simonet M, Sommer J, Gut A, Cerutti B, Van Der Vleuten CP, Dolmans D: **Impact of postgraduate training on communication skills teaching: A controlled study**. *BMC Medical Education* 2014, **14**(1):1-12.
28. Nasmith L, Franco ED: **Minor surgical procedures: Faculty development workshop**. *Canadian Family Physician* 1997, **43**(APR.):715-718.
29. Lemoine JB, Chauvin SW, Broussard L, Oberleitner MG: **Statewide interprofessional faculty development in simulation-based education for health professions**. *Clinical Simulation in Nursing* 2015, **11**(3):153-162.
30. Davis TS, Desselle SP: **Perceptions of a faculty cohort using education scholar as a basis for faculty development in active learning strategies**. *Currents in Pharmacy Teaching and Learning* 2013, **5**(5):394-401.
31. Sturpe DA, Layson-Wolf C, Lynn McPherson M, Haines ST: **An objective structured teaching examination (OSTE) for faculty development**. *Currents in Pharmacy Teaching and Learning* 2013, **5**(6):616-619.
32. Zagar M, Sampognaro L, Robertson C, Craft G: **A description and opinions of a longitudinal book club for comprehensive pharmacy faculty development**. *Currents in Pharmacy Teaching and Learning* 2019, **11**(9):909-914.
33. Thampy H, Bond K: **Sharing experiences of clinical teaching: An evaluation of contrasting workshops**. *Education for Primary Care* 2016, **27**(4):320-321.

34. Batsche CN, Monoson PK: **A FOUR-PHASE MULTIDISCIPLINARY FACULTY DEVELOPMENT MODEL IN AGING**. *Educational Gerontology* 1993, **19**(6):551-567.
35. Skeff KM, Stratos GA, Bergen MR: **Evaluation of a Medical Faculty Development Program: A Comparison of Traditional Pre/Post and Retrospective Pre/Post Self-Assessment Ratings**. *Evaluation & the Health Professions* 1992, **15**(3):350-366.
36. Delver H, Jackson W, Lee S, Palacios M: **FM POD: An evidence-based blended teaching skills program for rural preceptors**. *Family Medicine* 2014, **46**(5):369-377.
37. Gjerde CL, Kokotailo P, Olson CA, Hla KM: **A Weekend Program Model for Faculty Development with Primary Care Physicians**. *Family Medicine* 2004, **36**(SUPPL.):110-114.
38. Morzinski JA, Simpson DE: **Outcomes of a comprehensive faculty development program for local, full-time faculty**. *Family medicine* 2003, **35**(6):434-439.
39. Rust G, Taylor V, Herbert-Carter J, Smith QT, Earles K, Kondwani K: **The morehouse faculty development program: Evolving methods and 10-year outcomes**. *Family Medicine* 2006, **38**(1):43-49.
40. Williams BC, Schigelone AR, Fitzgerald JT, Halter JB: **Successful implementation of a faculty development program in geriatrics for non-primary care physician educators**. *Gerontology and Geriatrics Education* 2008, **28**(3):5-27.
41. Kuhnigk O, Schreiner J, Harendza S: **Sustained change in didactic skills--does teacher training last?** *GMS Zeitschrift für medizinische Ausbildung* 2013, **30**(3):Doc37-Doc37.
42. Garner SL, Killingsworth E, Bradshaw M, Raj L, Johnson SR, Abijah SP, Parimala S, Victor S: **The impact of simulation education on self-efficacy towards teaching for nurse educators**. *International Nursing Review* 2018, **65**(4):586-595.
43. Christie C, Bowen D, Paarmann C: **Effectiveness of faculty training to enhance clinical evaluation of student competence in ethical reasoning and professionalism**. *J Dent Educ* 2007, **71**(8):1048-1057.
44. Salerno SM, Malley PGO, Pangaro LN, Wheeler GA, Moores LK, Jackson JL: **Preceptor Improve Feedback in the Ambulatory Setting**. *J Gen Intern Med* 2002, **17**:779-787.
45. Kesselheim JC, Clayton CP, Fritz J, Smith RE, Gitlin SD, Reid E, Zuckerman KS, Kahn MJ: **The American Society of Hematology (ASH) Medical Educators Institute: a Pilot Faculty Development Project for Hematology Educators**. *Journal of Cancer Education* 2019, **34**(4):719-724.
46. Mokkapati A, Mada P: **Effectiveness of a teacher training workshop: An interventional study**. *Journal of Clinical and Diagnostic Research* 2018, **12**(2):JC09-JC12.
47. Blatt B, Plack MM, Simmens SJ: **Preparing interprofessional faculty to be humanistic mentors for medical students: The GW-gold mentor development program**. *Journal of Continuing Education in the Health Professions* 2018, **38**(2):117-125.
48. Arnett M, Korte D, Richards PS, Saglik B, Taichman LS, Kinney JS, Gwozdek AE: **Effect of Faculty Development Activities on Dental Hygiene Faculty Perceptions of and Teaching About Motivational Interviewing: A Pilot Study**. *Journal of Dental Education* 2017, **81**(8):969-977.

49. Gates P, Ubu N, Smithey L, Rogers J, Haden NK, Rodriguez T, Albino JEN, Evans C, Zarkowski P, Weinstein G *et al*: **Faculty Development for Underrepresented Minority Dental Faculty and Residents**. *Journal of Dental Education* 2013, **77**(3):276-291.
50. Johnstone-Dodge V, Bowen DM, Calley KH, Peterson TS: **A Faculty Development Course to Enhance Dental Hygiene Distance Education: A Pilot Study**. *Journal of Dental Education* 2014, **78**(9):1319-1330.
51. McAndrew M, Eidtson WH, Pierre GC, Gillespie CC: **Creating an Objective Structured Teaching Examination to Evaluate a Dental Faculty Development Program**. *Journal of Dental Education* 2012, **76**(4):461-471.
52. McAndrew M, Motwaly S, Kamens TE: **Long-Term Follow-Up of a Dental Faculty Development Program**. *Journal of Dental Education* 2013, **77**(6):716-722.
53. Sabato E, DeCastro JE, Fenesy K: **An Innovative, Comprehensive Faculty Recruitment and Development Program at One U.S. Dental School: Early Results**. *Journal of Dental Education* 2017, **81**(6):649-657.
54. Tricio JA, Montt JE, Ormeño AP, Del Real AJ, Naranjo CA: **Impact of Faculty Development Workshops in Student-Centered Teaching Methodologies on Faculty Members' Teaching and Their Students' Perceptions**. *Journal of Dental Education* 2017, **81**(6):675-684.
55. Berbano EP, Browning R, Pangaro L, Jackson JL: **The impact of the Stanford Faculty Development Program on ambulatory teaching behavior**. *Journal of General Internal Medicine* 2006, **21**(5):430-434.
56. Bigby J, Barnes HN: **Evaluation of a faculty development program in substance abuse education**. *Journal of General Internal Medicine* 1993, **8**(6):301-305.
57. Eckstrom E, Homer L, Bowen JL: **Measuring outcomes of a one-minute preceptor faculty development workshop**. *Journal of General Internal Medicine* 2006, **21**(5):410-414.
58. Green ML, Gross CP, Kernan WN, Wong JG, Holmboe ES: **Integrating teaching skills and clinical content in a faculty development workshop**. *Journal of General Internal Medicine* 2003, **18**(6):468-474.
59. Hatem DS, Barrett SV, Hewson M, Steele D, Purwono U, Smith R: **Teaching the medical interview: Methods and key learning issues in a faculty development course**. *Journal of General Internal Medicine* 2007, **22**(12):1718-1724.
60. Salerno SM, Jackson JL, O'Malley PG: **Interactive Faculty Development Seminars Improve the Quality of Written Feedback in Ambulatory Teaching**. *Journal of General Internal Medicine* 2003, **18**(10):831-834.
61. Sullivan AM, Lakoma MD, Billings JA, Peters AS, Block SD, Faculty PC: **Creating enduring change: demonstrating the long-term impact of a faculty development program in palliative care**. *Journal of general internal medicine* 2006, **21**(9):907-914.
62. Willett LR: **Brief report: Utilizing an audiotape for outpatient preceptor faculty development**. *Journal of General Internal Medicine* 2006, **21**(5):503-505.
63. Weppner WG, Fisher AK, Hagman M, Inouye L, King I, Smith CS, Wilper AP, Knight CL: **Taking SGIM's Teaching Educators Across the Continuum of Health Care on the Road-A Local, Interprofessional Faculty Development Innovation**. *Journal of graduate medical education* 2017, **9**(3):378-379.

64. Lang VJ, O'Connor AB, Blatt A, Gracey C: **Collaborative development of teaching scripts: An efficient faculty development approach for a busy clinical teaching unit**. *Journal of Hospital Medicine* 2012, **7**(8):644-648.
65. Mookherjee S, Monash B, Wentworth KL, Sharpe BA: **Faculty development for hospitalists: Structured peer observation of teaching**. *Journal of Hospital Medicine* 2014, **9**(4):244-250.
66. Podrazik PM, Levine S, Smith S, Scott D, DuBeau CE, Baron A, Whelan C, Johnson J, Cook S, Arora V *et al*: **The curriculum for the hospitalized aging medical patient program: A collaborative faculty development program for hospitalists, general internists, and geriatricians**. *Journal of Hospital Medicine* 2008, **3**(5):384-393.
67. Coogle CL, Hackett L, Owens MG, Ansello EF, Mathews JH: **Perceived self-efficacy gains following an interprofessional faculty development programme in geriatrics education**. *Journal of Interprofessional Care* 2016, **30**(4):483-492.
68. Koffel J, Reidt S: **An interprofessional train-the-trainer evidence-based practice workshop: Design and evaluation**. *Journal of Interprofessional Care* 2015, **29**(4):367-369.
69. Mkony CA, O'Sullivan PS, Owibingire SS, Fyfe MV, Omer S, Freeman P, Makubi A, Mloka DA, Portillo CJ, Leyna GH *et al*: **Teaching and educational scholarship in Tanzania: Faculty initiative to improve performance of health professions' students**. *Journal of Public Health Policy* 2012, **33**(SUPPL.1).
70. Sigalet EL, Davies JL, Scott EA, Brisseau GF, Shumway JB, Blackie BJ: **Designing interprofessional simulation based faculty development in a new women and children's hospital in the Middle East: A pilot study**. *Journal of Taibah University Medical Sciences* 2016, **11**(6):594-600.
71. Pinheiro SO, Liechty DK, Busch KV, Johnson ES, Dora DL, Butler RM: **Institutional impact of a part-time faculty development fellowship program for osteopathic community-based physicians**. *The Journal of the American Osteopathic Association* 2002, **102**(11):637-642.
72. Green ML: **A train-the-trainer model for integrating evidence-based medicine training into podiatric medical education**. *Journal of the American Podiatric Medical Association* 2005, **95**(5):497-504.
73. Baral N, Nepal AK, Paudel BH, Lamsal M: **Effect of teachers training workshop outcomes on real classroom situations of undergraduate medical students**. *Kathmandu University Medical Journal* 2015, **13**(50):162-166.
74. Baral N, Paudel BH, Das BKL, Aryal M, Gautam A, Lamsal M: **Preparing tutors for problem-based learning: An experience from B. P. Koirala Institute of Health Sciences, Nepal**. *Kathmandu University Medical Journal* 2010, **8**(29):141-145.
75. Yoon HB, Shin JS, Lee SH, Kim DH, Sung M, Amgalan N, Chinzorig T: **Transnational collaboration for faculty development in health professions education in Mongolia**. *Korean Journal of Medical Education* 2016, **28**(4):381-390.
76. Pololi LH, Frankel RM: **Humanising medical education through faculty development: Linking self-awareness and teaching skills**. *Medical Education* 2005, **39**(2):154-162.
77. Wong JG, Agisheva K: **Developing teaching skills for medical educators in Russia: A cross-cultural faculty development project**. *Medical Education* 2007, **41**(3):318-324.

78. Roos M, Kadmon M, Kirschfink M, Koch E, Jünger J, Strittmatter-Haubold V, Steiner T: **Developing medical educators - A mixed method evaluation of a teaching education program**. *Medical Education Online* 2014, **19**(1):1-8.
79. Smith MA, Cherazard R, Fornari A, Adelman P, Snopkowski M, Lesser M: **A unique approach to faculty development using an Observed Structured Teaching Encounter (OSTE)**. *Medical Education Online* 2018, **23**(1):1-5.
80. Schaefer EW, Wayne DB, McGaghie WC, Kozmic SE, Grais IM, Butter J: **Impact of Cardiac Physical Examination Faculty Development on Medical Student Performance: A Randomized Trial**. *Medical Science Educator* 2014, **24**(2):165-172.
81. Chung HS, Issenberg SB, Phrampus P, Miller G, Mo Je S, Ho Lim T, Min Kim Y: **The impact of an international faculty development program on simulation-based healthcare education**. *Medical Teacher* 2012, **34**(6):510-510.
82. Johansson J, Skeff K, Stratos G: **Clinical teaching improvement: The transportability of the Stanford Faculty Development Program**. *Medical Teacher* 2009, **31**(8).
83. Johansson J, Skeff KM, Stratos GA: **A randomised controlled study of role play in a faculty development programme**. *Medical Teacher* 2012, **34**(2).
84. Rodrigue C, Seoane L, Gala RB, Piazza J, Amedee RG: **Implementation of a faculty development curriculum emphasizing quality improvement and patient safety: Results of a qualitative study**. *Ochsner Journal* 2013, **13**(3):319-321.
85. Lee WW, Alkureishi ML, Isaacson JH, Mayer M, Frankel RM, London DA, Wroblewski KE, Arora VM: **Impact of a brief faculty training to improve patient-centered communication while using electronic health records**. *Patient Education and Counseling* 2018, **101**(12):2156-2161.
86. Perron NJ, Cullati S, Hudelson P, Nendaz M, Dolmans D, Van Der Vleuten C: **Impact of a faculty development programme for teaching communication skills on participants' practice**. *Postgraduate Medical Journal* 2014, **90**(1063):245-250.
87. Wong JG, Holmboe ES, Jara GB, Martin J, Becker WC, Fiellin DA: **Faculty development in small-group teaching skills associated with a training course on office-based treatment of opioid dependence**. *Substance Abuse* 2004, **25**(4):35-40.
88. Rosenbaum ME, Lenoch S, Ferguson KJ: **Outcomes of a teaching scholars program to promote leadership in faculty development**. *Teaching and Learning in Medicine* 2005, **17**(3):247-252.
89. Wong JG, Agisheva K: **Cross-cultural faculty development: Initial report of an American/Russian experience**. *Teaching and Learning in Medicine* 2004, **16**(4):376-380.
90. Wong JG, Fang Y: **Improving Clinical Teaching in China: Initial Report of a Multihospital Pilot Faculty Development Effort**. *Teaching and Learning in Medicine* 2012, **24**(4):355-360.
91. Phillips C, Bassell K, Fillmore L: **Transforming nursing education through clinical faculty development**. *Teaching and Learning in Nursing* 2019, **14**(1):47-53.
92. Liao KC, Pu SJ, Liu MS, Yang CW, Kuo HP: **Development and implementation of a mini-clinical evaluation exercise (mini-CEX) program to assess the clinical competencies of internal medicine residents: From faculty development to curriculum evaluation**. *BMC Medical Education* 2013, **13**(1):1-1.
93. Fornari A, Tortez LM, Lay M, Hirsch B, Tanzi D, Friedman I, Ricardo AP, Pekmezaris R, Branch W: **A mixed-methods approach to humanistic interprofessional faculty**

development. *Journal of Continuing Education in the Health Professions* 2018, **38**(1):66-72.

94. Moore JE, Pinsky MR: **Faculty development for fellows: Developing and evaluating a broad-based career development course for critical care medicine trainees**. *Journal of Critical Care* 2015, **30**(5):1152.e1151-1152.e1156.

95. Baker L, Egan-Lee E, Leslie K, Silver I, Reeves S: **Exploring an IPE faculty development program using the 3-P model**. *Journal of Interprofessional Care* 2010, **24**(5):597-600.

96. Efstathiou JA, Drumm MR, Paly JP, Lawton DM, O'Neill RM, Niemierko A, Leffert LR, Loeffler JS, Shih HA: **Long-term impact of a faculty mentoring program in academic medicine**. *PLoS ONE* 2018, **13**(11):1-12.

97. Safavi A, Sadeghi A: **Reconstructing Academic Staff Development Program: by Implementing a Supervised and Feedback on Experience Strategy in Order to Improve Their Teaching Skills in lesson Planning %J Bimonthly of Education Strategies in Medical Sciences**. 2017, **10**(5):358-363.

98. Windish DM, Gozu A, Bass EB, Thomas PA, Sisson SD, Howard DM, Kern DE: **A ten-month program in curriculum development for medical educators: 16 Years of experience**. *Journal of General Internal Medicine* 2007, **22**(5):655-661.

99. Derakhshan F, Allami A, Ahmadi S: **Effect of Faculty Training Programs on Improving Quality of Residency Exams in 2013-2014 %J Research in Medical Education**. 2015, **7**(1):19-26.

100. Sánchez-Mendiola M, Morales-Castillo D, Torruco-García U, Varela-Ruiz M: **Eight years' experience with a Medical Education Journal Club in Mexico: a quasi-experimental one-group study**. *BMC Medical Education* 2015, **15**(1):1-8.

101. Griffeth B, Wiederman M: **Faculty development effects on clerkship grades**. *Clinical Teacher* 2018, **15**(2):151-155.

102. Nemec EC, Welch B: **The impact of a faculty development seminar on the quality of multiple-choice questions**. *Currents in Pharmacy Teaching and Learning* 2016, **8**(2):160-163.

103. Dellinges MA, Curtis DA: **Will a Short Training Session Improve Multiple-Choice Item-Writing Quality by Dental School Faculty? A Pilot Study**. *Journal of Dental Education* 2017, **81**(8):948-955.

104. Tenzin K, Dorji T, Tenzin T: **Construction of multiple choice questions before and after an educational intervention**. *Journal of the Nepal Medical Association* 2017, **56**(205):112-116.

105. Schlair S, Dyche L, Milan F: **Longitudinal Faculty Development Program to Promote Effective Observation and Feedback Skills in Direct Clinical Observation**. *MedEdPORTAL : the journal of teaching and learning resources* 2017, **13**:10648-10648.

106. Register S, Peterson DT, Swatzell K, White ML: **Effect of interprofessional (IP) faculty development on perceptions of IP collaboration and on IP behaviors**. *Journal of Interprofessional Care* 2019, **33**(6):809-811.

107. McCurdy FA, Beck G, Maroon A, Gomes H, Lane PH: **The administrative colloquium: Developing management and leadership skills for faculty**. *Ambulatory Pediatrics* 2004, **4**(1 SUPPL.):124-128.

108. Rao SK, Carballo V, Cummings BM, Millham F, Jacobson JO: **Developing an Interdisciplinary, Team-Based Quality Improvement Leadership Training Program**

for Clinicians: The Partners Clinical Process Improvement Leadership Program. *American Journal of Medical Quality* 2017, **32**(3):271-277.

109. Yanamadala M, Criscione-Schreiber LG, Hawley J, Heflin MT, Shah BR: **Clinical Quality Improvement Curriculum for Faculty in an Academic Medical Center**. *American Journal of Medical Quality* 2016, **31**(2):125-132.

110. Baldwin CD, Gusic ME, Chandran L: **The Impact of a National Faculty Development Program Embedded Within an Academic Professional Organization**. *Academic Medicine* 2017, **92**(8):1105-1113.

111. Fleming GM, Simmons JH, Xu M, Gesell SB, Brown RF, Cutrer WB, Gigante J, Cooper WO: **A Facilitated Peer Mentoring Program for Junior Faculty to Promote Professional Development and Peer Networking**. *Academic Medicine* 2015, **90**(6):819-826.

112. Klein M, Niebuhr V, D'Alessandro D: **Innovative online faculty development utilizing the power of social media**. *Academic Pediatrics* 2013, **13**(6):564-569.

113. Grimes DA, Schulz KF, Droegemueller W, Munsick RA, Lisanti S: **A faculty development course in obstetrics and gynecology**. *American Journal of Obstetrics and Gynecology* 2000, **183**(4):1041-1044.

114. Jackevicius CA, Le J, Nazer L, Hess K, Wang J, Law AV: **A formal mentorship program for faculty development**. *American Journal of Pharmaceutical Education* 2014, **78**(5).

115. Reader S, Fornari A, Simon S, Townsend J: **Promoting Faculty Scholarship – An evaluation of a program for busy clinician-educators**. *Canadian Medical Education Journal* 2015, **6**(1):e43-e60.

116. John A, Joy TM, Paul N, Javed R, Sasidharan A, Menon UK: **Effectiveness of a 'Short training in teaching methodology' for entry level medical teachers**. *Indian Journal of Community Health* 2016, **28**(3):300-304.

117. Brown GE, Bharwani A, Patel KD, Lemaire JB: **An orientation to wellness for new faculty of medicine members: meeting a need in faculty development**. *International journal of medical education* 2016, **7**:255-260.

118. Srivastava TK, Waghmare LS, Rawekar A, Mishra VP: **Fostering educational research among medical teachers: Evaluation of a faculty development program in India**. *Journal of Clinical and Diagnostic Research* 2016, **10**(12):JC09-JC11.

119. Taylor CT, Berry TM: **A Pharmacy Faculty Academy to foster professional growth and long-term retention of junior faculty members**. *American journal of pharmaceutical education* 2008, **72**(2):32-32.
